# Simulation of Electrowetting-Induced Droplet Detachment: A Study of Droplet Oscillations on Solid Surfaces

**DOI:** 10.3390/ma16237284

**Published:** 2023-11-23

**Authors:** Nicolas T. Theodorou, Alexandros G. Sourais, Athanasios G. Papathanasiou

**Affiliations:** School of Chemical Engineering, National Technical University of Athens, 15780 Athens, Greece; nikolaostheodorou@mail.ntua.gr (N.T.T.); alexandrossourais@mail.ntua.gr (A.G.S.)

**Keywords:** electrowetting, droplet detachment, droplet oscillations, smart wetting, computational fluid dynamics, microfluidics, active materials

## Abstract

The electrowetting-induced detachment of droplets from solid surfaces is important for numerous applications in the fields of heat transfer and fluid mechanics. The forced oscillations of droplets on solid surfaces and their ability to detach are studied. In this study, the process is efficiently simulated by implementing a powerful methodology developed by our team. Our results agree with experiments showing that optimal detachment, in terms of actuation energy, is achieved when the application of voltage is synchronized with the spreading time of the droplet. Under these conditions, the droplet oscillates with a period close to that of a mirrored Rayleigh droplet. The relationship between the droplet’s oscillation period and its physical properties is examined. During voltage-droplet synchronization, the droplet’s ability to detach depends mostly on its contact angle, its viscosity, and the applied voltage. An energy analysis is also conducted, revealing how energy is supplied to the droplet by electrowetting-induced detachment.

## 1. Introduction

Droplet manipulation can be achieved by various methods, which fall into two main categories: active and passive. Active are the methods which require energy input (e.g., electrical energy—electrowetting, Kinetic Energy—and mechanical methods), while passive methods do not (e.g., microstructures or chemical substances on a surface that passively alter the droplet’s wettability). Among some examples of passive droplet manipulation are polymer-coated waterproof clothes [1] and atmospheric water harvesting (AWH) with specially structured surfaces [2]. Active droplet manipulation is found in fuel cells featuring vibration for water removal [3], reflective displays [4], and digital microfluidics (DMF) systems [5,6], with applications as lab-on-a-chip devices [7,8] and sensors [9,10]. Active manipulation of droplets enables continuous interaction with the droplet shapes and mobility on solid surfaces by altering the solid wettability in time. Among the available methods of active droplet manipulation, electrowetting (EW) is very efficient, and avoids the implementation of mechanical parts [11].

A typical electrowetting-on-dielectric (EWOD) setup involves the application of an electric potential between a conductive droplet and a substrate, which induces an attractive force at the droplet’s solid–liquid interface, leading to contact angle decrease and spreading of the droplet on the substrate. The electric potential difference is applied between a dielectric-coated metal substrate and the liquid (often using a submerged electrode inside the droplet). The dielectric coating ensures that the electric charges stay in place and the electric potential difference between the droplet and the substrate is maintained. An EWOD setup is like a parallel plate capacitor, where electric charges are stored into two parallel plates, which are separated by a dielectric material.

**Figure 1:**
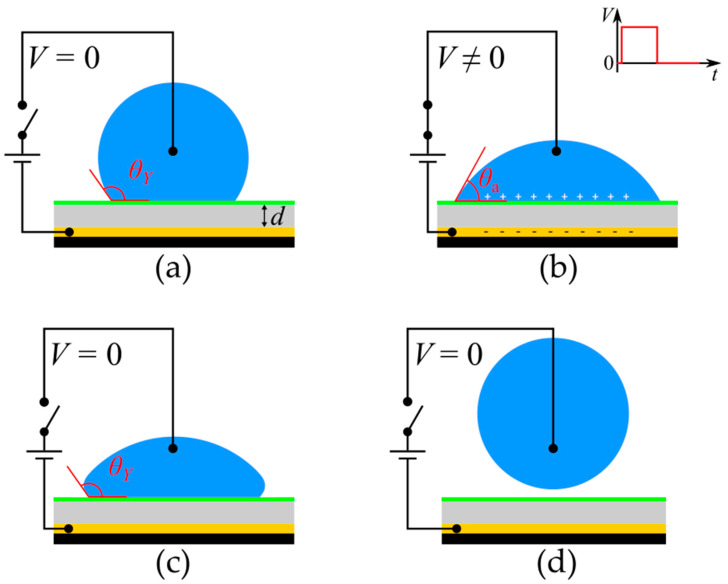
Electrowetting-induced spreading (**a**,**b**) and detachment (**c**,**d**) of a droplet. (**a**) Droplet resting on a solid substrate in the absence of voltage; the droplet forms a Young’s contact angle equal to *θ*_Y_. (**b**) Droplet spreads due to the application of voltage, forming a smaller apparent contact angle with the substrate (*θ*_a_ < *θ*_Y_). (**c**) Droplet starts to retract from its spread position when voltage is removed, and it regains its initial contact angle (*θ*_Y_). (**d**) Droplet detaches from the surface after its retraction in the absence of voltage.

While electrowetting intrinsically leads to attraction and the spreading of a droplet on a substrate, it can be suitably utilized to make a droplet detach from a surface by removing the applied voltage when the droplet is fully spread. This way, the contact angle immediately recovers its equilibrium value, *θ*_Y_, and the excess Surface Energy is converted to Kinetic Energy, forcing the droplet to recoil. Above a certain voltage threshold, the droplet can detach from the solid substrate. The process of electrowetting induced detachment is shown in Figure 1.

Droplet detachment from solid surfaces induced by electrowetting could contribute significantly to various technological applications. These applications include heat exchangers, where the removal of droplets from fins can increase heat transfer [12]; various microfluidic systems, some of which can be used for the construction of DNA microarrays [6,13]; and self-cleaning windshields [14].

**Figure 2:**
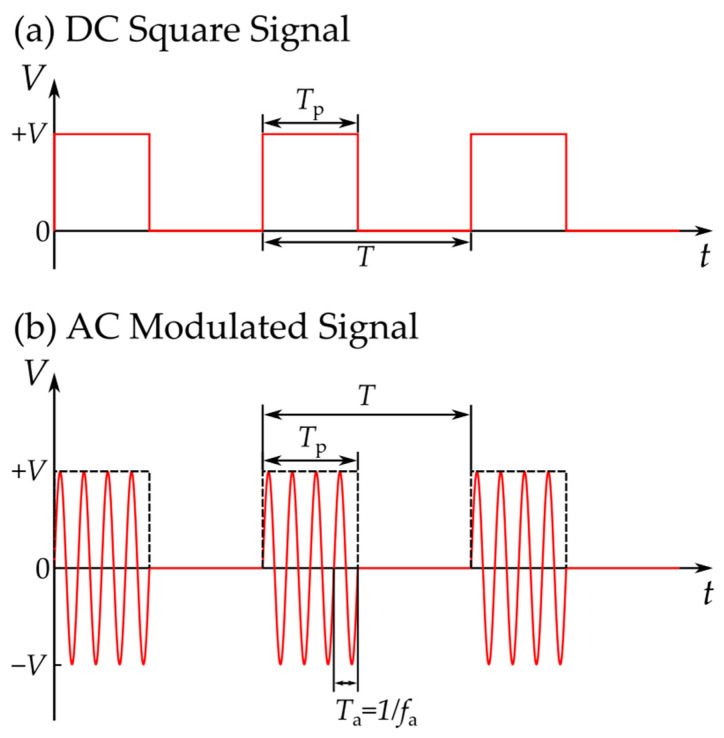
Schematic representation of (**a**) the DC square pulses and (**b**) the AC modulated signal used in EWOD setups for droplet detachment. The applied voltage of the signals is shown as a function of time. Both signals have a maximum amplitude of value +*V*, a period *T* and a pulse width *T*_p_. The AC signal has an oscillation period *T*_a_; which corresponds to its oscillation frequency *f*_a_.

In the process of electrowetting–induced droplet detachment, the applied voltage can have various waveforms. In most cases it is a couple of DC square pulses [11] or AC modulated signals [15,16]. DC square pulses can be applied once or continuously, with a defined application period (*T*). Each DC square pulse has a significant duration (width) (*T*_p_). In AC EW, signals of defined frequency (*f*_a_) are applied for a specific duration (*T*_p_). The signal Is reapplied after a certain period (*T*) has passed. AC signals are more efficient than DC signals, since their frequent oscillations can transfer higher amounts of energy to the droplets [16]. The voltage generated by a DC square signal and an AC modulated signal is shown as a function of time in Figure 2.

The optimum pulse width for droplet detachment has been investigated experimentally. Lee et al. have conducted experiments on water droplet detachment from hydrophobic surfaces using single DC voltage square pulses. They found that the optimal pulse duration, *T*_p_, for achieving a droplet detachment by applying the lowest possible voltage, is equal to the spreading time of the droplet *T*_s_ [11].

The spreading time of a droplet (*T*_s_) is defined as the time it takes for an initially resting droplet (before the application of voltage) to reach its maximum contact radius, when DC voltage is applied continuously. The application of voltage leads to the spreading of the droplet, up to a point where it starts to retract. After reaching a minimum contact radius, the droplet starts to spread again and oscillates over and over, until its excess energy dissipates by viscous forces, causing it to stay still at its new equilibrium position (which resulted from the application of voltage).

Wang et al. [16] built an experimental setup that detects whether a droplet spreads or recedes on a solid substrate, and accordingly applies voltage between the droplet and the substrate only during the spreading phase of the droplet. This setup continuously measures the variable (droplet shape dependent) capacitance of the capacitor formed by the droplet, the substrate, and their in-between dielectric layer, and thus indirectly estimates the droplet’s contact radius. The measured capacitance increases as the droplet’s footprint area increases. By applying voltage only during the spreading phase, the setup is able to achieve a trampolining behavior of the droplet, which oscillates with an increasing amplitude. Wang et al. have developed a simple oscillator model that estimates the various forms of energy that a droplet obtains as voltage is switched on and off [17].

In EW-induced detachment, it is important to synchronize the application of pulses with the spreading phase of the droplet, so that energy is transferred efficiently [11]. The resonant frequency of a droplet oscillating on a solid substrate depends on the droplet’s physical properties and its interactions with the substrate. Some examples of parameters that affect the droplet’s resonant frequency are droplet mass, surface tension, and Young’s contact angle (between the droplet and the substrate). Therefore, it is important to predict the resonance frequency and subharmonics of the droplet, and their dependence on its properties, so that EW-induced detachment can be performed in an optimal way. It is also important to predict the threshold voltage required for droplet detachment. Thus, the thorough investigation of the process of EW-induced detachment requires suitable theoretical/computational tools.

Several models [18,19,20,21] have been developed in the field of computational fluid dynamics (CFD) in order to simulate the EW-induced detachment of droplets from solid surfaces. Cavalli et al. [19] used a modified conventional hydrodynamic model. They used a relaxation factor alongside the no-slip boundary condition and a critical condition that describes the disconnection of the droplet’s contact line. Merdasi et al. [20] developed a VOF–CSF (volume of fluid–continuum surface force) model, which is able to simulate the interaction of droplets with heterogeneous substrates. Raman et al. [21] used a Lattice–Boltzmann (LB) method in order to describe the droplet’s interaction with the substrate.

Conventional hydrodynamic models present some disadvantages when it comes to modeling a droplet’s wetting and de-wetting dynamics. An explicit use of a no-slip boundary condition limits the movement of the droplet’s contact line (TPL). To cope with this, relaxation factors have been introduced, where the contact line of a droplet is simulated as a moving boundary, whose velocity is prescribed by theoretical correlations. Although this approach can predict the droplet’s TPL position on smooth surfaces, it is unable to describe scenarios with micro-structured surfaces, where multiple unknown contact lines appear. Diffuse-interface formulations, such as VOF models, can simulate the interaction of droplets with complex surfaces. However, these models have higher computational costs, due to the detailed meshes involved. Lattice–Boltzmann (LB) simulations introduce extremely high computational costs when used in the scale lengths of actual droplets (mm). Therefore, they are considered unsuitable for our system.

Chamakos et al. [22] recently developed a continuous level, sharp-interface model that is capable of simulating droplet spreading dynamics over complex structured surfaces. This is achieved by introducing the solid–liquid interactions as a disjoining pressure term. This model has already been used to successfully simulate the electrowetting-induced droplet detachment from smooth or structured surfaces [18,23], while keeping the computational costs at low levels [22]. Most importantly, this model does not suffer from the drawbacks and limitations of the other approaches that were previously described.

In the present study, the detachment of droplets from solid surfaces with the assistance of electrowetting was simulated, using the disjoining pressure hydrodynamic model. Several scenarios were simulated, in which DC square pulses of different durations and voltages were applied to droplets of different volumes, in order to verify whether the optimum pulse duration coincides with the droplets’ spreading time, and to validate how well the simulation results matched the experiments [11]. Another set of simulations was executed, in which DC voltage was applied between a droplet and a substrate during the droplet’s spreading phases by monitoring its contact radius, similar to the trampolining droplet setup that uses capacitive sensing [16]. These simulations were conducted for various droplet volumes, densities, surface tensions, viscosities, applied voltages, and contact angles. The goal of this work was to determine how these parameters affect the droplet’s oscillation dynamics, as well as its ability to detach. An energy analysis was also performed, to better understand how the application of voltage leads to droplet detachment. An overarching objective is to develop a versatile and reliable computational tool for the rational design of EW based processes; this is why emphasis is placed on comparisons against experiments.

## 2. Materials and Methods

### 2.1. Hydrodynamic Model

We implemented our model in COMSOL Multiphysics^®^ software package (version 6.1). The governing equations of the hydrodynamic model are the Navier–Stokes equations, which describe the conservation of momentum and mass:

(1) $$\rho \left(\frac{\partial u}{\partial t}+u\cdot \nabla u\right)=-\nabla P+\mu \nabla^{2}u+G\;\;\;\;\;\;\;\;\;\;\nabla \cdot u=0$$

where *ρ* is the density of the droplet; ***u***, the velocity field; *t*, the time; *P*, the pressure; *μ*, the dynamic viscosity; and, ***G***, the gravitational field. ***G*** = *ρ**g***, where ***g*** is the vector of gravitational acceleration with a magnitude of |***g***| = 9.81 m/s^2^ [18,22].

In our simulations, a disjoining pressure term was introduced in the Young–Laplace equation to describe the interactions between the droplet and the solid substrate. This term was incorporated in the balance of normal stresses, which was applied as a boundary condition at the droplet’s surface. It is described by the following equation:

(2) $$\tau_{\mathrm{nn}|\mathrm{ambient}}-\tau_{\mathrm{nn}|\mathrm{liquid}}+\Delta P-\gamma_{\mathrm{LA}}C-P_{\mathrm{LS}}=0$$

where *τ*_nn|liquid_ and *τ*_nn|ambient_ are the normal viscous stresses at the surface of the droplet by the liquid and the ambient phase, respectively [18,22]; Δ*P* is the pressure difference between the concave surface (droplet) and the convex surface (ambient phase); $\gamma_{\mathrm{LA}}$ is the interfacial tension coefficient between the droplet and the ambient phase (e.g., air); *C* is the droplet’s mean curvature; and *P*_LS_ is the disjoining pressure that describes the solid–liquid interactions. This equation is a modification of the Young–Laplace equation.

Our modelling assumes the existence of a thin layer of air between the liquid and the solid surface. For the calculation of disjoining pressure an expression similar to a Lennard–Jones potential was used, which includes a short range repulsive and a long-range attractive term.

(3) $$\frac{R_{0}}{\gamma_{\mathrm{LA}}}P_{\mathrm{LS}}=w^{\mathrm{LS}}\left[{\left(\frac{\sigma }{\delta /R_{0}+\varepsilon }\right)}^{C_{1}}-{\left(\frac{\sigma }{\delta /R_{0}+\varepsilon }\right)}^{C_{2}}\right]$$

The term *w*^LS^ in the equation above is the well depth and can be correlated to the Young’s contact angle:

(4) $$w^{\mathrm{LS}}=\frac{\left(C_{1}-1\right)\left(C_{2}-1\right)\left(1+\cos\theta_{\Upsilon }\right)}{\sigma \left(C_{1}-C_{2}\right)}$$

*R*_0_, is the characteristic length of the droplet (radius of a sphere with the same volume) and *θ*_Υ_ is the Young’s contact angle between the droplet and the substrate in the absence of electric field. The exponents *C*_1_ and *C*_2_ are parameters that affect the range of the repulsive and the attractive areas of the potential; *δ* is the Euclidean distance between a point on the droplet’s surface and the substrate (width of thin air layer); *σ* and *ε* are additional dimensionless parameters that affect the form of the potential, whose difference is equal to the minimum available distance that can appear between the droplet and the substrate [22]:

*δ*_min_ = *R*_0_ (*σ* − *ε*) (5)

After fitting the above parameters (*C*_1_, *C*_2_, *σ*, *ε*) to experimental data on water droplet/PTFE substrate systems [22,24], the values used for the estimation of interactions between the droplets and the solid substrate are: *C*_1_ = 12, *C*_2_ = 10, *σ* = 0.009, *ε* = 0.008.

Apart from the liquid–solid interactions and the normal stresses applied at the liquid–ambient interface, friction is also applied at the surface of the droplet as a form of tangential stress. Friction is a result of the substrate’s roughness, and is present even on smooth surfaces, since nano-scale heterogeneities still exist due to the arrangement of molecules that form the solid surface.

The friction is introduced into the model as a Navier slip boundary condition applied at the solid–liquid interface:

*τ*_nt|friction_ = *β*_eff_ (***t*** · ***u***) (6)

where *τ*_nt|friction_ is the friction tangential stress, *β*_eff_ is an effective slip coefficient, and ***t*** is the unit tangential vector.

This model is able to estimate the dynamic contact angle hysteresis of a sliding droplet and can therefore successfully describe the generated friction at the surface of droplets, even on inclined substrates, with great consistency with experimental results [25].

The coefficient *β*_eff_ is active only in the vicinity of the solid–liquid interface [23,25]:

(7) $$\beta_{\mathrm{eff}}=\beta_{\mathrm{SL}}\frac{\mu }{R_{0}}\left(1-\tanh\left[p_{\mathrm{trs}}\left(\frac{\delta }{\delta_{\min}}-1\right)\right]\right)$$

where *β*_SL_ is the dimensionless inverse slip length and *p*_trs_ is a parameter that controls the transition between the solid–liquid and the liquid–ambient interface, respectively.

In Section 3.1, the values of the parameters used for water droplets are consistent with our previous works [25], *β*_SL_ = 1000 and *p*_trs_ = 5. In rest of the tested scenarios (Section 3.2, Section 3.3 and Section 3.4), a value of *β*_SL_ = 0 was used.

**Figure 3:**
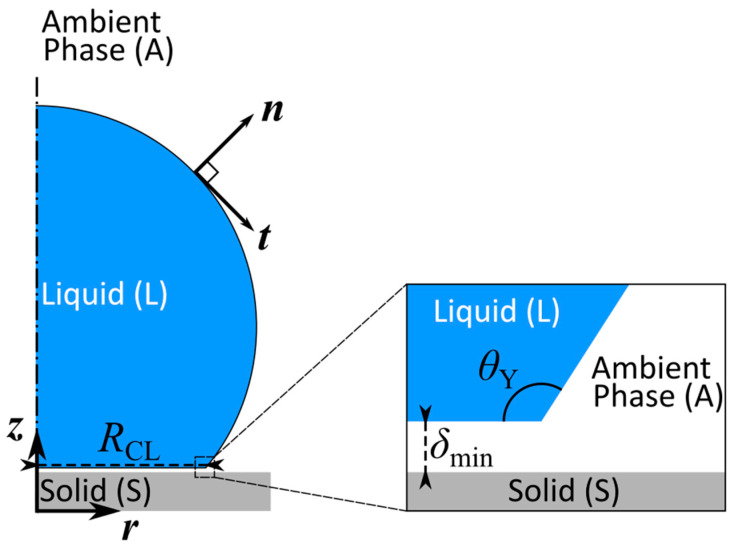
Schematic representation of a droplet equilibrating on a flat solid substrate in a 2D axis-symmetric coordinate system.

In the executed simulations, droplets were modeled as axisymmetric. An axisymmetric droplet is shown in Figure 3.

### 2.2. Accounting for the Electric Pulses

Electric square pulses are incorporated into the numerical model as a modification of the droplet’s contact angle for a certain amount of time (pulse duration), according to Lippmann’s equation [26]:

(8) $$\cos\theta_{a}=\cos\theta_{Y}+\frac{\varepsilon_{0}\;\varepsilon_{r}\;V^{2}}{2d\gamma_{\mathrm{LA}}}=\cos\theta_{Y}+\eta$$

where *θ*_a_ is the apparent contact angle when voltage, *V*, is applied; *θ*_Y_ is the Young’s contact angle between the droplet and the substrate without any electric fields (e.g., 116°); *d* the width of the dielectric layer between the droplet and the substrate’s electrodes (e.g., 5 μm); *ε*_0_ the vacuum permittivity (8.854 × 10^−12^ F/m); *ε*_r_ the relative permittivity of the solid below the droplet (e.g., 3.14); $\gamma_{\mathrm{LA}}$ the water–air interfacial tension (0.072 N/m); and $\eta =\frac{\varepsilon_{0}\varepsilon_{r}V^{2}}{2d\gamma_{LA}}$ is the electrowetting number. The example values shown correspond to experiments [11].

In this work, electric square pulses were incorporated into the system’s boundary conditions by modifying the droplet’s contact angle (*θ*) and setting it equal to the apparent one that is derived from Lippman’s equation (*θ*_a_) during pulse applications. In this approach, droplet conductivity is assumed to be large enough for the system’s response to be independent of it. In experiments, this is ensured by the addition of an electrolyte (e.g., NaCl) [11]. Charges inside the droplet move practically instantaneously in comparison to the time scales of motion of the droplet. All of the simulations were executed at the initial frame of a droplet freely standing in the absence of an electric field.

#### 2.2.1. Application of Single Square Pulses

In the simulations presented in Section 3.1, where single square pulses were applied, the results of which were compared to those of experiments, the contact angle in the boundary equations (*θ*(*t*)) was set equal to the apparent one (*θ*_a_) for times less than or equal to the pulse duration (*T*_p_), while, for the rest of the simulation, it was equal to the Young’s contact angle (*θ*_Y_).

*θ*(*t*) = *θ*_a_, *t* < *T*_p_ *θ*(*t*) = *θ*_Y_, *t* ≥ *T*_p_ (9)

#### 2.2.2. Application of Continuous Voltage

In order to find the spreading time of sessile droplets that are reported in Section 3.1, simulations were executed, where the contact angle of the droplets was set as equal to the apparent one (*θ*_a_) throughout the whole simulation.

*θ*(*t*) = *θ*_a_ (10)

#### 2.2.3. Application of Square Pulses Synchronized with the Droplet’s Spreading Phase

In the rest of our simulations, presented in Section 3.2, Section 3.3 and Section 3.4, electric square pulses were applied during the spreading phase of the droplet, and were removed during the retraction phase. This was accomplished by monitoring the velocity of the droplet’s three-phase contact line (TPL). When the droplet spreads, the direction of its TPL’s velocity points towards the outside of the droplet (*u*_CL_ > 0), and while it retracts the TPL’s vector points towards the inside of the droplet (*u*_CL_ < 0).

The position of the droplet’s contact line (TPL) was determined in our simulations by finding the maximum value of the radial coordinate (*r* coordinate in a 2D axisymmetric system) within the droplet’s surface, observed below a critical height of *z*_crit_ = 1.5 *δ*_min_.

*R*_CL_ = max(*r*),       *r*,*z* ∈{ droplet surface | *z* < *z*_crit_ } (11)

Due to fluctuations of the TPL with time, an average of different estimations was used that were calculated using slightly different critical heights.

The velocity of the droplet’s TPL was calculated as the time derivative of the droplet’s TPL position:

(12) $$u_{\mathrm{CL}}=\frac{dR_{\mathrm{CL}}}{dt}$$

As previously mentioned, the droplet’s TPL fluctuates with time. In order to avoid extreme values in velocity caused by this fluctuating behavior, the derivative was calculated using a time-step equal to or greater than Δ*t*_min_ = 0.5 ms. The calculated velocities were also smoothed using a moving average of 10 different values. Due to the existence of the droplet’s higher oscillation modes (other than the main one) or even numerical noise, the velocity of its TPL can change sign very frequently, giving the false impression that the droplet spreads and retracts many times within a short period of time, while, in reality, it is constantly spreading or retracting. In order to cope with this behavior, a time delay (Δ*t*_crit_), equal to 0.1 ms for contact angles (*θ*_Y_) of 90–125° and 0.5 ms for contact angles of 135–150°, was introduced when estimating the droplet’s spreading or retraction phase, based on the sign of its TPL velocity. While the droplet was considered to be spreading, voltage was applied by setting the droplet’s contact angle, equal to the apparent one (*θ*_a_). While the droplet was considered to be retracting, voltage was removed by setting the actual contact angle equal to the Young’s contact angle (*θ*_Y_). Therefore, pulses were applied as follows:

Spreading: *u*_CL_ > 0 for Δ*t* > Δ*t*_crit_, *θ*(*t*) *= θ*_a_ Retraction: *u*_CL_ < 0 for Δ*t* > Δ*t*_crit_, *θ*(*t*) *= θ*_Y_ (13)

### 2.3. Energy Calculations

In order to perform an energy analysis of the electrowetting-induced droplet detachment, various contributions to the energy were calculated according to the expressions explained below.

According to the energy balance, the system’s total energy must be maintained:

(14) $$E_{\mathrm{total}}=E_{\mathrm{total}}^{\mathrm{initial}}=E_{\mathrm{total}}^{\mathrm{final}}\Leftrightarrow E_{\mathrm{total}}=E^{\mathrm{initial}}=E^{\mathrm{final}}+E_{\mathrm{output}}-E_{\mathrm{input}}$$

where $E_{\mathrm{total}}^{\mathrm{initial}}$ and $E_{\mathrm{total}}^{\mathrm{final}}$ are the initial and final values of the total energy of the system comprising the droplet and its surroundings. This remains constant and equal to *E*_total_. $E^{\mathrm{initial}}$ and $E^{\mathrm{final}}$ are the initial and final energy inside the droplet, while *E*_input_ and *E*_output_ are the amounts of energy that have entered and exited the droplet, during the transition from the initial to the final state.

The energies inside the droplet, have the form of Kinetic, Gravitational, and Surface Energy, while the energy exiting the droplet is Viscous Dissipation (transferred to its environment as heat) and the energy entering the droplet is the Electric Work provided to the droplet. Therefore, the energy balance takes the following form:

(15) $$E_{\mathrm{total}}=K^{i}+U_{G}^{i}+E_{S}^{i}=K^{f}+U_{G}^{f}+E_{S}^{f}+E_{V}-W_{\mathrm{el}}$$

where *Κ*, *U*_G_, and *E*_S_ are the droplet’s Kinetic Energy, Gravitational Potential Energy, and Surface Energy, respectively. Their initial and final values are marked with the superscripts ^i^ and ^f^, respectively. *E*_V_ is the Accumulated Viscous Dissipation energy that has exited the droplet and *W*_el_ is the Accumulated Electric Work that has been given to the droplet.

The droplet’s Kinetic Energy is calculated [18] as follows:

(16) $$K={\int_{\Omega }\frac{1}{2}\rho \left|u\right|}^{2}d\Omega$$

where *ρ* is the droplet’s density, **u** is its velocity at a point in space, and Ω is the computational domain (volume), in which the droplet is present.

The droplet’s Gravitational Potential Energy is equal to:

(17) $$U_{G}=\int_{\Omega }\rho gh\;d\Omega$$

where *h* is the elevation of a point inside the droplet, in reference to the substrate’s surface; and *g* = 9.81 m/s is the earth’s gravitational acceleration constant.

In general, the droplet’s Surface Energy is calculated [18] as follows:

(18) $$E_{S}=\gamma_{\mathrm{LS}}A_{\mathrm{LS}}+\gamma_{\mathrm{SA}}A_{\mathrm{SA}}+\gamma_{\mathrm{LA}}A_{\mathrm{LA}}$$

where $\gamma_{\mathrm{LS}}$, $\gamma_{\mathrm{SA}}$, and $\gamma_{\mathrm{LA}}$ are the liquid–solid, solid–ambient phase, and liquid–ambient phase surface tension coefficients, respectively, while *A*_LS_, *A*_SA_, and *A*_LA_ are the surface areas of the liquid–solid, solid–ambient phase, and liquid–ambient phase interface, respectively.

At equilibrium, the different surface tension coefficients are related, according to the Young’s equation:

(19) $$\gamma_{\mathrm{SA}}=\gamma_{\mathrm{LS}}+\gamma_{\mathrm{LA}}\cos(\theta_{Y})$$

where *θ*_Y_ is the Young’s contact angle between the droplet and the substrate, in the absence of voltage.

By using the Surface Energy *E*_S,F_ of a free solid surface *A*_SA,F_ as a reference, the Surface Energy can be calculated as follows:

(20) $$E_{S}=\gamma_{\mathrm{LS}}A_{\mathrm{LS}}+\gamma_{\mathrm{SA}}\left(A_{\mathrm{SA}}-A_{\mathrm{SA},F}\right)+\gamma_{\mathrm{LA}}A_{\mathrm{LA}}+E_{S,F}$$

The sum of the surface areas of the solid-ambient and liquid–solid interfaces must equal the total surface area of the solid.

(21) $$A_{\mathrm{LS}}+A_{\mathrm{SA}}=A_{\mathrm{SA},F}$$

By combining Equations (19)–(21), the droplet’s Surface Energy can be expressed as:

(22) $$E_{S}=A_{\mathrm{LA}}\gamma_{\mathrm{LA}}-A_{\mathrm{LS}}\gamma_{\mathrm{LA}}\cos(\theta_{Y})+E_{S,F}$$

The droplet’s surface energy can be renormalized in reference to the initial Surface Energy $E_{S}^{i}$ of the resting sessile droplet and then subtracted by the resting droplet’s Gravitational Energy $U_{G}^{i}$, such that the total energy at *t* = 0 is zero ($E_{\mathrm{total}}=U_{G}^{i}+{E_{S}^{i}}^{\prime }=0\;J$).

(23) $$\begin{array}{l} {E_{S}}^{\prime }=A_{\mathrm{LA}}\gamma_{\mathrm{LA}}-A_{\mathrm{LS}}\gamma_{\mathrm{LA}}\cos(\theta_{Y})+E_{S,F}-E_{S}^{i}-U_{G}^{i}\Leftrightarrow \\ {E_{S}}^{\prime }=\left(A_{\mathrm{LA}}-A_{\mathrm{LA}}^{i}\right)\gamma_{\mathrm{LA}}-\left(A_{\mathrm{LS}}-A_{\mathrm{LS}}^{i}\right)\gamma_{\mathrm{LA}}\cos(\theta_{Y})-U_{G}^{i} \end{array}$$

where $A_{\mathrm{LA}}^{i}$ and $A_{\mathrm{LS}}^{i}$ are the surface areas of the liquid–ambient phase and liquid–solid interface of the initially resting droplet.

According to Lippmann’s approach the droplet, under the influence of an electric field, forms a capacitor with the substrate. The energy of the formed capacitor can be calculated as [26]:

(24) $$E_{\mathrm{el}}=C\frac{V^{2}}{2}=\eta \cdot \gamma_{\mathrm{LA}}A_{\mathrm{LS}}\Leftrightarrow E_{\mathrm{el}}=\left(\cos\theta_{a}-\cos\theta_{Y}\right)\gamma_{\mathrm{LA}}A_{\mathrm{LS}}$$

where $C=\varepsilon_{r}\varepsilon_{0}\frac{A_{\mathrm{LS}}}{d}$ is the capacitance of the formed droplet–substrate capacitor.

The work given to the droplet during the application of each pulse can be calculated as the difference of the actual capacitor energy, minus the capacitor energy at the beginning of pulse application.

(25) $$W_{\mathrm{el},P}=E_{\mathrm{el}}-E_{\mathrm{el},P0}=\eta \cdot \gamma_{\mathrm{LA}}\left(A_{\mathrm{LS}}-A_{\mathrm{LS},P0}\right)$$

where *E*_el,P0_ and *A*_LS,P0_ are the capacitor energy and the surface area of the droplet’s solid–liquid interface at the beginning of pulse application, respectively.

When no pulse is applied at the droplet, the electric work given is zero. The Accumulated Electric Work (*W*_el_) given to the droplet at a time *t*, can be calculated as a sum of the work given by each pulse, up to the considered point in time. If *N* pulses have been applied, with the last being still active, and if the capacitor energy at the end of application of each one of the previous pulses is *E*_el,*I*_, the Accumulated Electric Work can be calculated as follows:

(26) $$W_{\mathrm{el}}(t)=\sum_{P=1}^{N}W_{\mathrm{el},P}(t)=\sum_{i=1}^{N-1}\left(E_{\mathrm{el},i}-E_{\mathrm{el},P0,i}\right)+\left(E_{\mathrm{el},N}(t)-E_{\mathrm{el},P0,N}\right)$$

The Viscous Dissipation per time and unit volume of the droplet, in its axisymmetric form, is expressed as [18,27]:

(27) $$\varphi \equiv \tau :\nabla u=2\mu \left[{\left(\frac{\partial u_{r}}{\partial r}\right)}^{2}+{\left(\frac{u_{r}}{r}\right)}^{2}+{\left(\frac{\partial u_{z}}{\partial z}\right)}^{2}+\frac{1}{2}{\left(\frac{\partial u_{z}}{\partial r}+\frac{\partial u_{r}}{\partial z}\right)}^{2}\right]$$

where ***τ*** is the stress tensor, ∇u is the strain-rate tensor, and τ:∇u is the double dot product operation between the two tensors.

The Accumulated Viscous Dissipation of the droplet can be calculated by integrating the above quantity over time and the computational domain forming the droplet.

(28) $$E_{V}=\int_{t}\int_{\Omega }\varphi \;d\Omega dt$$

## 3. Results and Discussion

### 3.1. Optimal DC Square Pulse Width for Droplet Detachment—Model Validation

In the first part of our study, we simulated the detachment of droplets from solid surfaces using single DC square pulses and compared our results with published experimental results. Lee et al. reported that the optimal duration of a single DC square pulse for droplet detachment is equal to the spreading time of the droplet [11].

Several scenarios of electrospreading were examined, where constant voltages of different amplitudes were applied. This way, the dependence of the spreading time on the applied voltage was obtained. Simulations were performed to obtain the dependence of the minimum electrowetting number required for droplet detachment (threshold electrowetting number) on the duration of a DC pulse. Then, the optimum pulse width was determined by detecting the pulse duration that corresponds to the lowest threshold electrowetting number. The procedure described above was performed for droplets of two different volumes: *V*_D_ = 0.4 μL and *V*_D_ = 5 μL. We selected the same conditions with the ones of the experiments (*θ*_Y_ = 116°, *ρ* = 1000 kg/m^3^, *μ* = 1.005 mPa·s, $\gamma_{\mathrm{LA}}$ = 0.072 N/m) [11]. The friction between the droplet and the substrate was simulated with a factor of *β*_SL_ = 1000, which is used to recover the no-slip boundary condition [25]. The Young–Lippmann equation for a simple needle electrode setup was used to correlate the electrowetting numbers, i.e., the applied voltages, with the apparent contact angles.

**Figure 4:**
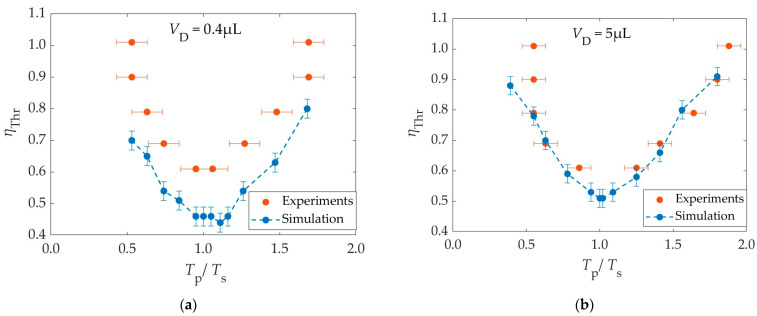
The threshold electrowetting number required for droplet detachment as a function of the dimensionless pulse width (*T*_p_/*T*_s_) of the applied DC square pulse. Droplet volumes: (**a**) 0.4 μL, (**b**) 5 μL. Blue: simulation, orange: experiments [11]. *T*_s_ is the spreading time corresponding to the overall threshold electrowetting number for detachment of the droplet of the considered size.

The spreading times (*T*_s_) of the 0.4 μL and the 5 μL droplet were found to be *T*_s_ = 1.9 ms and *T*_s_ = 6.4 ms, respectively, with an error of ±0.5 ms. These values are very close to the ones experimentally reported by Lee et al. [11] (i.e., 2 ms and 7 ms, respectively). The spreading time is insensitive to the electrowetting number, as reported by Lee et al. [11]. In Figure 4, the threshold electrowetting number (*η*_Thr_) is shown as a function of the dimensionless pulse width (*T*_p_/*T*_s_) for two droplet volumes. The experimental values published by Lee et al. [11] are also plotted for comparison.

In the simulation results, an error bar in the value of the threshold EW number has been drawn, which corresponds to an error of ±2° in the apparent contact angle (*θ*_A_). The error bar was added, as there is a range of EW numbers, corresponding to Δ*θ*_A_ = 2°, where the droplet detaches for some EW numbers but does not detach for others.

Ie smallest among the minima of the electrowetting numbers was found to be *η*_Thr_* = 0.46 (*θ*_A_ = 89°) in the case of 0.4 μL and *η*_Thr_* = 0.51 (*θ*_A_ = 86°) in the case of 5 μL. In both cases, the overall minimum of the electrowetting number corresponds to a pulse width (presented as normalized with the spreading time, *T*_s_) which is practically equal to the spreading time, i.e., *T*_p_/*T*_s_ = 1. Therefore, it is confirmed that the optimal pulse width is equal to the spreading time.

The results of the simulations wer” ver’ close to the experimental ones overall. In the case of 5 μL, the two sets match within their error bars and agree both in terms of optimal pulse duration, as well as threshold voltage. A small deviation appears at very high EW numbers (*η*_Thr_ > 0.85), due to contact angle saturation, which leads to deviation from Lippmann’s equation (saturation area: 0.76 ≤ *η* ≤ 0.99) [11]. In the case of 0.4 μL, the threshold EW numbers in the simulations are consistently smaller than the experimental ones, by an amount of Δ*η* ≃ 0.15, leading to easier detachment. This is probably caused by the presence of the needle electrode in the experiments that leads to energy dissipation. This effect is larger for smaller droplets. However, both the simulation and the experiments agree in terms of optimal pulse duration.

**Figure 5:**
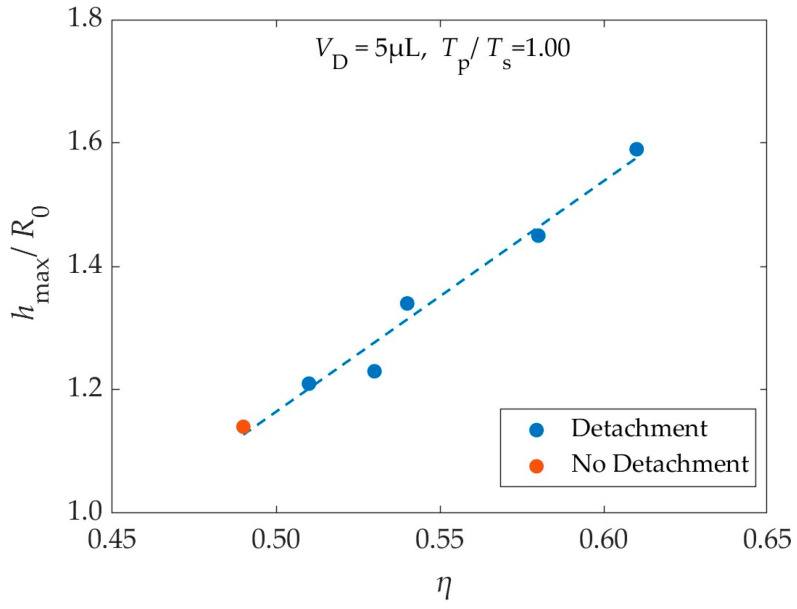
Maximum dimensionless height (*h*_max_/*R*_0_) reached by the 5 μL droplet as a function of the electrowetting number (*η*) of the applied pulse, as it resulted from the simulations. Blue points: cases where the droplet detaches from the substrate; orange points: cases where the droplet does not detach from the substrate.

As the simulations showed, in most cases, by applying a higher voltage (i.e., higher, *η*) while maintaining the pulse width (*T*_p_), the droplet detaches more easily and jumps higher. This behavior is displayed in Figure 5, where the dimensionless maximum height (*h*_max_/*R*_0_) is plotted as a function of the electrowetting number (*η*). The maximum vertical displacement of the center of mass is defined as the h_max_, and here is normalized with, *R*_0_.

As shown in Figure 5, for EW numbers close to threshold, the droplet’s highest position is almost a linear function of the EW number. This behavior is reasonable, since, according to Equation (25), the work (*W*_el_) that is given to droplets of the same size (similar footprint area) via the application of a single pulse is proportional to the EW number. This work becomes Gravitational Potential Energy. Therefore, the maximum elevation reached by the droplet is proportional to the EW number.

**Figure 6:**
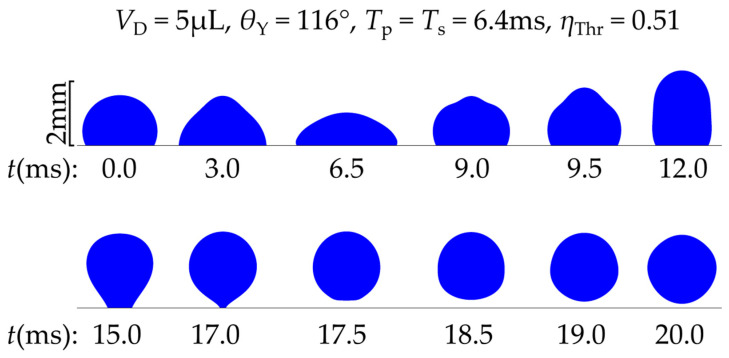
Shape and vertical position of a 5 μL water droplet as a function of time, according to the simulations. The droplet is initially resting on a smooth solid surface with a Young’s contact angle of 116°. A single DC square pulse of threshold voltage (*η*_Thr_ = 0.51) is applied between the droplet and the substrate at *t* = 0. The pulse width is equal to the spreading time of the droplet *T*_p_ = *T*_s_ = 6.4 ms.

Figure 6 shows the computed evolution of the droplet’s shape and position.

In Figure 6, it can be seen that, under the application of voltage the droplet spreads and when voltage is removed, the droplet regains its initial contact angle (*θ*_Y_) and retracts. At the end it detaches from the substrate. The simulation’s results show remarkable resemblance to the droplet’s pictures in experiments [11]. The detachment of the droplet is also shown in Video S1 of the Supplementary Materials.

### 3.2. Parameters That Affect the Oscillation Frequencies of a Droplet during Electrowetting-Induced Detachment

In this second part of our study, we conducted a parametric analysis in order to examine the dependence of the oscillation frequency of the droplet’s center of mass on the droplet’s mass (*m*), surface tension (*γ*), electrowetting number (*η*), viscosity (*μ*), and Young’s contact angle (*θ*_Υ_). In these simulations, the droplet was stimulated by the application of DC square pulses, synchronized with its spreading phase. This was achieved by monitoring the velocity of the droplet’s TPL, as described in the Section 2. Thus, the duration and timing of the pulses was optimal in terms of achieving droplet detachment.

**Figure 7:**
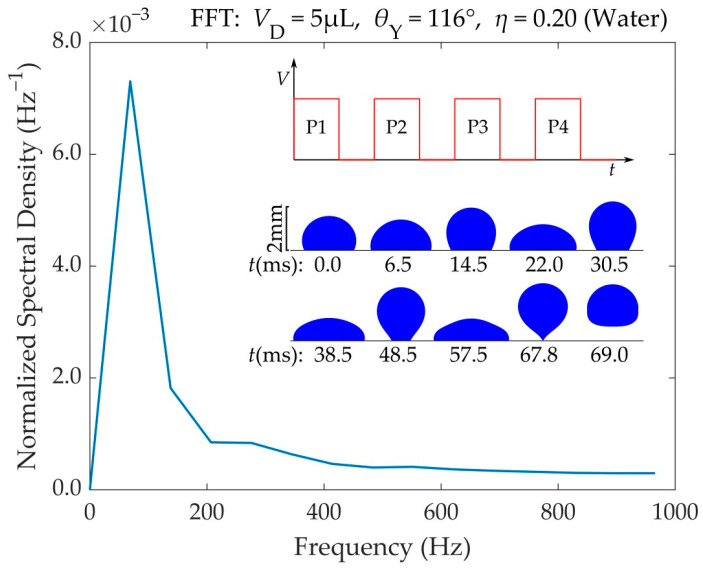
Range of frequencies for the center of mass vertical oscillation of a 5 μL water droplet prior to its detachment. This droplet detaches after the application of four consecutive DC square pulses (P1–P4) of *η* = 0.20. The pulse duration (*T*_p_) was adjusted to match the droplet’s spreading time (*T*_s_) in each oscillation by monitoring the droplet’s TPL velocity. The liquid (water) had a viscosity of *μ* = 1 mPa·s, and formed a contact angle with the substrate equal to *θ*_Y_ = 116°. There was no friction between the droplet and the substrate (*β*_SL_ = 0).

The application of voltage pulses makes the droplet oscillate on the substrate. In this situation, the droplet’s center of mass oscillates vertically. A Fast Fourier Transform (FFT) of the droplet’s center of mass vertical displacement reveals the range of frequencies characterizing the droplet’s center of mass oscillation. Figure 7 shows the spectrum of frequencies for a water droplet that detaches after the application of four consecutive pulses. The FFT was performed with Matlab^®^. A frequency of *f* = 69 Hz, corresponding to an oscillation period of *T* = 14.5 ms, is dominant. This period is roughly twice the droplet’s spreading time (*T*_s_ = 6.4 ms), mentioned in Section 3.1. The detachment of the droplet is also shown in Video S4 of the Supplementary Materials.

Our simulations showed that the solid–liquid friction coefficient (*β*_SL_) has no effect on the droplet’s oscillation frequency. Thus, the rest of our study is based on scenarios where the friction between the droplet and the substrate was absent (*β*_SL_ = 0, free slip). It must be noted though, that for high viscosities (*μ* ≥ 5.00 mPa·s) an increase in the friction coefficient (*β*_SL_), increases the threshold EW number (*η*_Thr_), making detachment more difficult.

#### 3.2.1. Effect of Mass and Surface Tension on the Droplet’s Oscillation Period

Two different sets of simulations were used, in order to examine the dependence of the actuated droplet’s center of mass oscillation period on the droplet’s mass. In the first one, the density of the droplet was always the same (*ρ* = 1000 kg/m^3^), while different droplet volumes (*V*_D_ = 0.4, 1, 3, 5, 10 μL) were studied. In the second one, the droplet’s volume was kept constant (*V*_D_ = 5 μL), while different densities (*ρ* = 100, 500, 1000, 2000 kg/m^3^) were examined. In all of these simulations, square pulses that correspond to an EW number of *η* = 0.33 were applied.

All droplets detached from the substrate during their second oscillation and reached similar heights. This is visible in their Gravitational Potential Energy at maximum elevation, which scales almost linearly with *V* and *ρ* (Supplementary Materials–Sections S.3.3 and S.3.4). Therefore, droplet mass does not seem to affect the droplet’s ability to detach.

In order to examine the dependence of the droplet’s center of mass oscillation period on its surface tension, the droplet’s mass was kept constant, while different surface tension coefficients (*γ*) were tested. In every case, pulses of *η* = 0.33 were being applied, while the Young’s contact angle was equal to *θ*_Y_ = 116°. In all scenarios (except the case where *γ* = 18 mN/m) the droplet detached from the substrate during its second oscillation. Generally, we found that the higher the surface tension the easier the droplet detachment and the higher the maximum jumping height. This can also be seen in the droplets’ Gravitational Potential Energy at maximum elevation, which scales linearly with *γ*^0.75^ (Supplementary Material—Section S.3.5). Therefore, there is a slight dependence of the droplet’s ability to detach on its surface tension.

The different oscillation mode “of” free droplet have been studied by Lord Rayleigh [28] and Sir Horace Lamb [29]. According to their theory, the oscillations of a free droplet are mainly affected by the droplet’s inertia and surface tension (liquid–vapor). The oscillation frequencies of each mode are described by the Rayleigh–Lamb equation:

(29) $$f_{n}=\frac{1}{T_{n}}=\sqrt{\frac{n\left(n-1\right)\left(n+2\right)\gamma }{3\pi \rho V}}$$

where *f_n_* (*T_n_*) is the droplet’s oscillation frequency (period) that corresponds to a selected mode *n*, *γ* is the droplet’s surface tension coefficient, *ρ* is its density, and *V* is its volume. For free oscillating droplet *n* ≥ 2. The dominant oscillation mode, in which the amplitude is the largest and the frequency is the smallest, corresponds to *n* = 2.

In the case of a sessile droplet on a solid substrate, capillary interaction forces between the droplet and the substrate are present and affect the droplet’s oscillations. If significant viscous forces are present, they also affect the oscillations of the droplet. If the droplet is in the underdamped regime, the oscillations are mainly affected by the droplet’s inertia and capillary forces, while, if the droplet is in an overdamped condition, the droplet’s oscillation timescales are dominated by the viscous forces [30].

It has been reported that the oscillation period of an Inviscid sessile droplet on a solid surface with which the droplet forms a contact angle of 90° is practically the same as the oscillation period of a mirrored Rayleigh droplet [31]. The oscillation period of the mirrored Rayleigh droplet results from the Rayleigh–Lamb equation, when a volume twice the actual volume of the droplet is inserted.

(30) $$T_{n}=\sqrt{\frac{3\pi \rho V}{n\left(n-1\right)\left(n+2\right)\gamma }}\;\;\;\overset{n=2}{\underset{V=2V_{D}}{\Rightarrow }}\;\;\;T_{2M}=\sqrt{\frac{3\pi \rho V_{D}}{4\gamma }}$$

where *T*_2M_ is the oscillation period of a mirrored Rayleigh droplet, oscillating in its second mode (*n* = 2—dominant oscillation). *V*_D_ is the actual volume of the sessile droplet.

**Figure 8:**
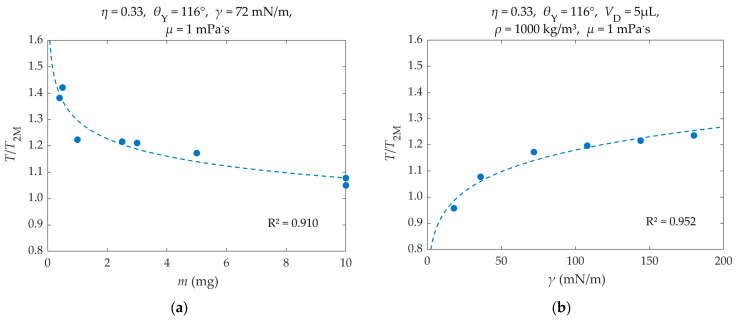
Dimensionless oscillation period (*T*/*T*_2M_) of the droplet’s center of mass, as a function of (**a**) droplet mass (*m*) and (**b**) surface tension coefficient (*γ*). The simulation results (dots) have been fitted with Equation (31) (dashed lines). R^2^ is the coefficient of determination of the fit.

In Figure 8, the dimensionless droplet oscillation period (*T/T*_2M_)—actual dominant period *T* of the droplet’s center of mass from FFT divided by the period of the mirrored Rayleigh droplet *T*_2M_—is plotted as a function of the droplet’s mass (*m*) and its surface tension coefficient (*γ*). A good fit of the simulation results is provided by Equation (31). According to the fitting, the droplet’s dimensionless oscillation period is a power law function of its mass and surface tension.

(31) $$\frac{T}{T_{2M}}=a\cdot m^{b}\cdot \gamma^{c}\;\;\;\;\;\;\;\mathrm{where}:\;a=0.829,\;b=-0.08,\;c=0.105,\;m\;\mathrm{in}\;\mathrm{mg},\;\gamma \;\mathrm{in}\;\mathrm{mN}/m$$

As Figure 8 shows, in most cases the droplet’s center of mass oscillation period is close to that of the mirrored Rayleigh droplet (*T*/*T*_2M_ is close to 1). Deviations appear for small droplet masses (0.4–0.5 mg), where the resulting oscillation periods are larger. This behavior also appears at high surface tension coefficients (*γ* > 72 mN/m).

The mirrored Rayleigh droplet’s oscillations should match the oscillations of an inviscid droplet that forms a contact angle of *θ*_Υ_ = 90°, in the absence of friction between the droplet and the substrate or of any applied voltage [31]. Therefore, the deviations of the simulated scenarios are due to the presence of viscosity and an obtuse contact angle (*θ*_Υ_ = 116° > 90°), which also changes when voltage is applied.

As the droplet’s mass gets smaller, the presence of other forces (e.g., viscous forces, droplet–interfacial forces) become more significant due to the droplet’s smaller inertia, thus slowing the droplet down and increasing its oscillation period.

By increasing the liquid–ambient phase surface tension coefficient, while maintaining the Young’s contact angle, the interfacial tension coefficients between the other phases are altered as well, according to the Young’s equation—Equation (19). Therefore, with an increase in the surface tension coefficient, the surface forces between the droplet and the ambient phase and between the droplet and the substrate become larger. The latter increases the droplet’s center of mass oscillation period.

Overall, the droplet’s center of mass oscillation period seems to be approximately proportional to the square root of the droplet’s mass and its inverse surface tension coefficient, according to the mirrored Rayleigh droplet.

#### 3.2.2. Effect of Contact Angle, Electrowetting Number, and Viscosity on the Droplet’s Oscillation Period

**Figure 9:**
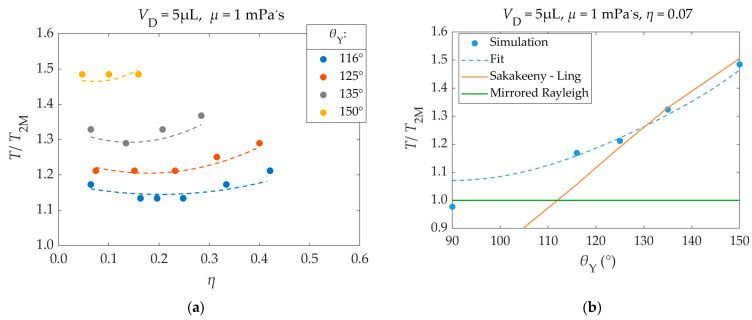
(**a**): Dimensionless oscillation period (*T*/*T*_2M_) of a 5 μL water droplet’s center of mass, as a function of the EW number (*η*) for different Young’s contact angles (*θ*_Y_). Points: results; dashed lines: fitting—Equation (32). (**b**): Dimensionless oscillation period of the droplet, as a function of the Young’s contact angle (*θ*_Y_). A square electric pulse of *η* = 0.07 is applied during the droplet’s spreading phases. Blue dots: simulation results; blue dashed line: fitting—Equation (32); orange line: Sakakeeny and Ling’s model results [32]; green line: mirrored Rayleigh droplet (*T*/*T*_2M_ = 1.0). In all cases *T*_2M_ = 12.8 ms and *β*_SL_ = 0.

Here, the dependence of the droplet’s oscillation period, prior to detachment, on its Young’s contact angle (*θ*_Υ_), EW number (*η*), and viscosity (*μ*) was examined. The results are shown in Figure 9. In all cases, the droplet oscillated at least twice, prior to detachment. The droplet’s oscillation period (*T*) was obtained by an FFT. In all scenarios, the FFT was applied on the droplet’s center of mass trajectory during its first oscillation, regardless of the total number of oscillations, for consistency.

Young’s contact angle (*θ*_Υ_), electrowetting number (*η*), and viscosity (*μ*) are all parameters that affect the droplet’s ability to detach from the substrate. This is further analyzed in Section 3.3.

The computational results in Figure 9 have been fitted with the following equation, which follows their behavior very closely (Mean Absolute Percentage Error (MAPE) = 1.05% in the points of Figure 9a):

(32) $$T/T_{2M}=\left[a{\left(\eta -\eta_{b}\right)}^{2}+b\right]\cdot {\left(\theta_{\Upsilon }-90{}^{\circ}\right)}^{2}+c\;\;\;\mathrm{where}\;\eta_{b}=D{\left(\theta_{\Upsilon }-90{}^{\circ}\right)}^{2}+E\cdot \mu +F$$

and *a* = 1.23 × 10^−3^ deg^−2^, *b* = 1.09 × 10^−4^ deg^−2^, *c* = 1.07, *D* = −4.48 × 10^−5^ deg^−2^, *E* = 8.03 × 10^−3^ (mPa·s)^−1^, *F* = 0.225.

Young’s contact angle plays an important role in the droplet’s oscillation period. For a contact angle of 90°, the droplet’s oscillation period can be estimated using the mirrored Rayleigh droplet [31]. The oscillations of such droplets have also been analyzed by Lyubimov et al. [33,34]. However, for different contact angles the droplet deviates from the mirrored Rayleigh behavior.

Bostwick and Steen developed a model for predicting the different oscillation modes of droplets with an acute contact angle (*θ*_Y_ ≤ 90°). Their results are available in lookup tables [31]. Sakakeeny and Ling developed a numerical model that can predict the oscillation frequency of droplets for different Bond numbers and different contact angles, ranging from 50° up to 150° [32].

The simulation results in Figure 9b were compared against the predictions of Sakakeeny and Ling’s model. For a better comparison, the smallest tested EW number (*η* = 0.07) is considered, since Sakakeeny and Ling’s model does not account for the application of voltage. The results for different EW numbers are shown Figure S1d of the Supplementary Material. Sakakeeny and Ling’s model results were calculated for a 5 μL water droplet (*ρ* = 1000 kg/m^3^, *γ* = 72 mΝ/m), under the influence of earth’s surface gravitational acceleration (*g* = 9.81 m/s^2^). The parameters and fitting functions used in this model are obtained from data for 50° ≤ *θ*_Y_ ≤ 150° [32].

For high contact angles (125°–150°), the simulation results are almost identical with those of Sakakeeny and Ling’s model [32]. As the contact angle gets closer to 90°, the oscillation periods come closer to those of the mirrored Rayleigh droplet, as expected [31], while deviating from Sakakeeny and Ling’s model. This behavior of the oscillation period was approximated with a parabolic dependence on its contact angle in Equation (32) (Τ/Τ_2M_ ∝ (*θ*_Υ_–90°)^2^, applicable for contact angles ≥90°).

According to Lippmann’s approach—Equation (8)—which was used in the simulations, the application of voltage temporarily modifies the droplet’s contact angle. Therefore, it affects the droplet’s oscillations, since it alters the surface interaction forces near the droplet’s three-phase contact line (TPL).

As shown in Figure 9a, a change in the EW number has a small effect on the droplet’s oscillation period. Dash et al. [35,36] reported, based on experiments, that the spreading time of a droplet during DC EW actuation is independent of the applied voltage. Wang et al. [17] reported, based on theoretical publications, that the resonance frequency of a droplet in an EWOD setup scales with the EW number as:

(33) $$\omega \propto {\left[\gamma R_{0}{}^{-3}\left(6-\eta \right)/\left(3\rho_{w}+2\rho_{a}\right)\right]}^{1/2}$$

where *ω* is droplet’s resonance frequency, *R*_0_ its equivalent radius, *η* is the EW number, *ρ*_w_ is the density of the droplet, and *ρ*_a_ is the density of the ambient phase.

The simulation results were compared to this model. The comparison is shown in the Supplementary Material (Section S1). Both the simulation and the theoretical model [17] indicate that, for high EW numbers, the droplet’s oscillation period increases with an increase in the EW number. However, for smaller EW numbers, simulations show that the oscillation period initially decreases with an increase in the EW number, forming a local minimum (around *η*_b_ in Equation (32)—*T*/*T*_2M_ ∝ (*η* − *η*_b_)^2^). This behavior could be a result of the apparent contact angle (*θ*_a_) approaching 90° for these small EW numbers.

In Figure 9a, as the Young’s contact angle increases, the number of data points becomes smaller. This is a result of the droplet detaching more easily in the case of large Young’s contact angles, prior to completing one whole oscillation on the substrate when high EW numbers are used. Under these conditions, the droplet’s oscillation period cannot be properly determined. The resulting oscillation periods from the simulation of such scenarios were significantly larger than the rest, and they were considered outliers. Thus, they were not included in the figure.

The simulations concerned droplets of different viscosities, ranging from 1 to 10 mPa·s. The results showed no significant dependence of the droplet’s oscillation period on its viscosity. This behavior is expected, based on studies by Vo et al. [30], since the droplet was in the underdumped regime. In all cases, there was no friction between the droplet and the substrate and the droplet overshot its equilibrium radius while oscillating. A numerical model by Hong et al. [36] also predicts that the oscillation period of the high viscosity droplet (10 mPa·s) is larger than that of the low viscosity droplet (1 mPa·s), only by 0.2%. This complies with the simulations’ results, as it shows that, within the tested range, an increase in the droplet’s viscosity causes a negligible increase in its oscillation period. In Figure 9, all the results correspond to a viscosity of 1 mPa·s. The results of droplets of different viscosities (5 mPa·s, 10 mPa·s), as well as a detailed presentation of the theoretical models, is available in the Supplementary Material (Section S1).

### 3.3. Parameters That Affect the Droplet’s Ability to Detach

The Young’s contact angle (*θ*_Υ_), the EW number (*η*), and viscosity (*μ*) are parameters that strongly affect the droplet’s ability to detach. In this section, we quantify the droplet’s ability to detach, in terms of the number of required pulses.

When the energy provided to the droplet is not sufficient for the latter to detach using a single pulse, the droplet might be able to detach after the application of multiple pulses. In this case, the droplet’s oscillation amplitude increases, as the droplet overshoots its equilibrium contact radius more and more with each pulse application. Hence, when multiple pulses are applied, the threshold EW number is lower, in comparison to the case of a single pulse application.

**Figure 10:**
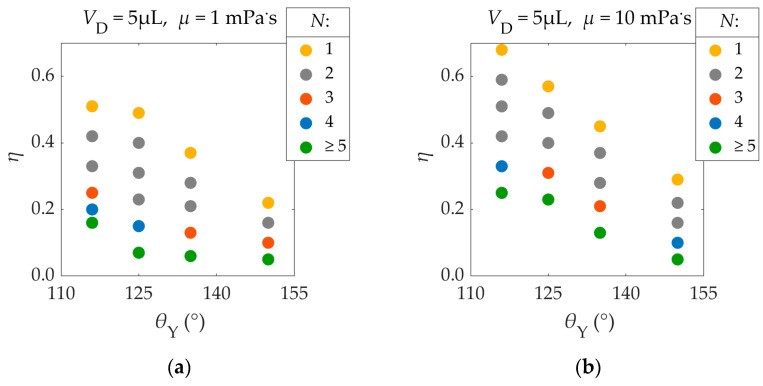
Number of voltage pulses (*Ν*) that are required for the detachment of a 5 μL droplet, as a function of the Young’s contact angle (*θ*_Y_) and the EW number (*η*), for different viscosities (*μ*): (**a**) *μ* = 1 mPa·s, (**b**) *μ* = 10 mPa·s. The density, surface tension, and solid–liquid friction coefficient of the droplet are *ρ* = 1000 kg/m^3^, $\gamma_{\mathrm{LA}}$ = 72 mN/m, and *β*_SL_ = 0, respectively.

This behavior is shown in Figure 10, where the number of required pulses (*N*) for droplet detachment is displayed as a function of the Young’s contact angle (*θ*_Υ_) and the EW number (*η*). Pulses were synchronized with the droplets’ spreading phase.

As shown in Figure 10, by decreasing the EW number, the number of required pulses (*N*) increases. For example, in the case of Figure 10a, where *θ*_Y_ = 125°, the application of a single pulse (*N* = 1) of *η* = 0.49 leads to droplet detachment. When the EW number is lowered to *η* = 0.40, two pulses (*N* = 2) are required for detachment. If the EW number is lowered to *η* = 0.15, four pulses are required for detachment (*N* = 4). A further reduction of the EW number to *η* = 0.07 does not lead to droplet detachment after four pulse applications (*N* ≥ 5). Therefore, by increasing the number of electric pulses, the threshold EW number and voltage required for detachment are lowered. The same behavior is observed in experiments [11].

However, achieving detachment using three or more pulse applications is difficult. For small EW numbers, like the ones in the cases of three or more pulse applications, the droplet’s ability to detach is very sensitive on the EW number. Thus, a very small range of EW numbers can be used in order to detach the droplet with a specific number of applied pulses. For example, in one case of Figure 10a where *θ*_Y_ = 125°, a reduction of the EW number from *η* = 0.4 to *η* = 0.23 does not affect the droplet’s ability to detach, as two pulse applications are required. When the EW number is further reduced to *η* = 0.15, droplet detachment requires four pulses. Hence, the range of EW numbers that lead to detachment after three pulse applications, if existent, is smaller than Δ*η* = 0.23 − 0.15 = 0.07, while, in the case of two pulse applications, it is at least larger than Δ*η* = 0.4 − 0.23 = 0.17. For very small EW numbers, detachment is non-feasible, even after infinite pulse applications. This is mainly caused by energy dissipation, as well as small errors in the synchronization of the pulses with the droplet’s spreading phase that accumulate with each pulse application.

Figure 10 also shows that, the higher the Young’s contact angle (*θ*_Υ_), the easier it is for the droplet to detach from the substrate (smaller *η*). Furthermore, when the viscosity (*μ*) of the fluid is larger, Viscous Dissipation increases, making it harder for the droplet to detach (higher *η*).

The displayed results can be useful for the design of real-life applications of EW-induced droplet detachment. Achieving detachment with three or more oscillations can be difficult in experiments, due to the reasons mentioned above. Therefore, the use of single or double electric square pulses is recommended, unless contact angle saturation is still present. In all of the executed simulations, the EW numbers were below the contact angle saturation value (*η* < 0.75) [11]. The results for a droplet with a viscosity of 5 mPa·s are presented in the Supplementary Material (Section S2).

It must be noted that, in the cases of large viscosity (*μ* ≥ 5 mPa·s), if the solid–liquid friction coefficient (*β*_SL_) is increased, the threshold EW number (*η*_Thr_) increases as well. This is not the case when *μ* = 1 mPa·s, as energy dissipation is generally small regardless of *β*_SL_.

The detachment of droplets in the case of *μ* = 1 mPa·s and *θ*_Y_ = 116° is shown in Videos S1–S4 of the Supplementary Materials, for *N* = 1 (*η* = 0.51), *N* = 2 (*η* = 0.33), *N* = 3 (*η* = 0.25), and *N* = 4 (*η* = 0.20), respectively.

### 3.4. Energy Analysis

In order to better understand the phenomenon of electrowetting-induced detachment and the way in which energy is being transferred inside the droplet during the application of voltage, an energy analysis was performed. Part of this analysis is briefly presented in this section, while the rest is available at the article’s Supplementary Material (Section S3).

**Figure 11:**
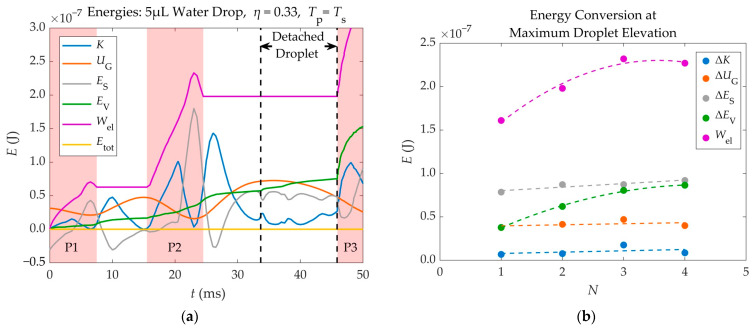
(**a**) Various forms of a 5 μL droplet’s energy, during EW induced detachment, as a function of time. The energies shown are Kinetic Energy (*K*), Gravitational Potential Energy (*U*_G_), Surface Energy (*E*_S_), Accumulated Viscous Dissipation (*E*_V_), Accumulated Electric Work (*W*_el_), and Total Energy (*E*_tot_). Two pulses of *η* = 0.33 are applied prior to detachment. The application times of the pulses (P1, P2, P3) are marked as pink regions in the background. Two pulses (P1, P2) are applied prior to detachment, while a third one (P3) is automatically applied after the droplet reattaches to the substrate. (**b**) Energy gains at the maximum elevation of a 5 μL droplet, as a function of the number of applied pulses (*N*) prior to detachment. The gains of each energy are equal to the difference of the energy value at maximum elevation minus the value corresponding to the initially resting droplet. The EW numbers were the same for all pulses in each scenario and equal to *η* = 0.51, 0.33, 0.25, and 0.20 for *N* = 1, 2, 3, and 4, respectively. The droplets are made of water (*μ* = 1 mPa·s, *ρ* = 1000 kg/m^3^, *γ* = 72 mN/m), while there is no friction between the droplet and the substrate (*β*_SL_ = 0).

The different energies involved in the detachment of a 5 μL water droplet from a substrate of *θ*_Y_ = 116°, using two (*N* = 2) consecutive electric pulses of *η* = 0.33, synchronized with its spreading phase, are shown in Figure 11a, as a function of time. These include Kinetic Energy (*K*), Gravitational Potential Energy (*U*_G_), Surface Energy (*E*_S_), Electric Work (*W*_el_), and Viscous Dissipation (*E*_V_).

As shown in Figure 11a, the electric work provided to the droplet during the application of voltage, is continuously converted to other forms of energy. For instance, the Gravitational Energy reaches its maximum and minimum values at the highest and lowest position of each oscillation, respectively. The Surface Energy reaches its maximum value when the droplet is fully spread, and its minimum value when the droplet reaches its initial footprint during retraction, since its shape is closest to that of the resting droplet. The Kinetic Energy approaches zero at the lowest and highest points of each oscillation, where the droplet is almost motionless. The Accumulated Viscous Dissipation only increases with time. Viscous Dissipation increases significantly every time a voltage pulse is applied, especially when voltage is removed or when the droplet reattaches to the substrate, as its shape changes rapidly.

Due to the synchronization between the electric pulses and the droplet’s spreading phase, the electric power is mostly positive, as the electric force that drives the droplet’s spreading has the same direction with the velocity of the spreading droplet’s TPL (they both point towards the outside of the droplet). Some delays in the deactivation of the simulated pulses can cause the electric power to become negative. In these cases, the droplet starts to retract while voltage is still applied and the electric force pulling the droplet points oppositely to the droplet’s TPL velocity. This is depicted in Figure 11a, as peaks in the curve of the Accumulated Electric Work (*W*_el_), where the latter drops for a short period of time. The Accumulated Electric Work increases in the form of steps, where each step corresponds to an applied pulse.

An energy analysis was also made on four different scenarios, where a 5 μL water droplet detached after the application of *N* = 1, 2, 3, and 4 pulses that corresponded to EW numbers equal to *η* = 0.51, 0.33, 0.25, and 0.20, respectively. In each scenario, all pulses were of the same EW number. In all scenarios, the Young’s contact angle was equal to 116°. This analysis was made in order to determine the most efficient method for detaching the droplet, from an energy perspective, among the use of a single pulse or multiple pulses. The Electric Work given to the droplet in each scenario, as well as the different energy forms that it gets converted to at the droplet’s maximum elevation, are shown in Figure 11b.

The energy analysis of Figure 11b shows that, when the 5 μL droplet gets barely detached from the substrate, its Kinetic, Gravitational, and Surface Energy (useful forms of energy) at the maximum elevation reached, are independent of the number of pulses (*N*) that are used in the detachment process. However, the Electric Work and the Viscous Dissipation inside the droplet become larger as the number of pulses (*N*) increases.

Therefore, although using more pulses reduces the threshold EW number and the required voltage, it increases the energy required for droplet detachment. This means that the more pulses are used for detachment, the less efficient the process is, as more energy is used, while the droplet reaches the same height after detachment. Hence, it is recommended to detach the droplet using one pulse, unless contact angle saturation is present.

The detachment of the droplets, analyzed in Figure 11, is shown in Videos S1–S4 of the Supplementary Material, for *N* = 1, *N* = 2, *N* = 3, and *N* = 4, respectively.

### 3.5. Summary of the Effect of Different Parameters on the Droplet’s Oscillation Period and Ease of Detachment

After having studied the impact of mass (*m*), surface tension coefficient (*γ*), Young’s contact angle (*θ*_Y_), EW number (*η*), and viscosity (*μ*) on the droplet’s oscillation period and its ability to detach, a summary was made.

The effect of each tested parameter on the droplet’s oscillation period and its ability to detach is summarized in Table 1.

## 4. Conclusions

Our model was able to successfully simulate the process of EW-induced droplet detachment from solid surfaces, matching results from experiments [11].

The simulations confirmed that the most efficient pulse width for the detachment of a droplet using a DC square pulse is equal the spreading time of the droplet. Therefore, the use of capacitive sensors, in order to match these two times by monitoring the droplet’s contact radius, is a good method for achieving droplet detachment. Our simulations were also able to simulate the “trampolining” behavior of a droplet on a solid substrate, under the effect of consecutive voltage pulse applications.

The oscillation frequency of a droplet that moves on a solid substrate during the process of EW-induced detachment, when pulses synchronized with the droplet’s spreading phase are applied, was found to be close to that of a mirrored Rayleigh droplet, especially if the contact angle between the droplet and the substrate is near 90°. The period of oscillations (*T*) appeared to be almost proportional to (*m*/*γ*)^½^, where *m* is the mass of the droplet and *γ* is its surface tension coefficient. The oscillation period was studied for different values of Young’s contact angles (*θ*_Υ_), EW numbers (*η*), and viscosities (*μ*), and correlations between the period and these parameters were developed, taking into account previous work in the literature.

A change in mass (*m*) and surface tension (*γ*) of the droplet did not seem to significantly affect its ability to detach. However, decreasing the EW number (*η*), Young’s contact angle (*θ*_Υ_), or increasing the droplet’s viscosity (*μ*) makes detachment harder and increases the number of voltage pulses (*N*) that are required for detachment. It was confirmed that by increasing the number of applied pulses, the threshold EW number decreases. The number of required pulses (*N*) for droplet detachment, according to the simulations, has been presented for different values of the Young’s contact angle (*θ*_Υ_), EW number (*η*), and viscosity (*μ*). These results can be useful for conducting experiments.

Lastly, an energy analysis of the simulation results was performed in order to examine the amount of work that is given to the droplet during the process of EW-induced detachment and the way in which it gets converted into different forms of the droplet’s energy. The energy analysis showed that, although by applying more pulses the threshold EW number for droplet detachment is reduced, the process becomes less efficient and demands more energy, due to the higher levels of Viscous Dissipation.

## Figures and Tables

**Table 1:** The effect of mass (*m*), surface tension coefficient (*γ*), Young’s contact angle (*θ*_Y_), EW number (*η*), viscosity (*μ*), and solid–liquid friction coefficient (*β*_SL_) on the droplet’s oscillation period and its ability to detach, according to the simulations.

Parameter	Impact on Oscillation Period (*T*)	Observed Relation with Oscillation Period (*T*)	Affects Droplet’s Ability to Detach	Easier Detachment
Mass (*m*)	Very Large	*T* ∝ $\sqrt{\frac{m}{\gamma }}$	Not Significantly	-
Surface Tension (*γ*)	Very Large	*T* ∝ $\sqrt{\frac{m}{\gamma }}$	Yes, Small Impact	With increasing *γ*
Young Contact Angle (*θ*_Υ_)	Large	*T* ∝ (*θ*_Υ_–90°)^2^, *θ*_Y_ ≥ 90°	Yes	With increasing *θ*_Y_
Electrowetting Number (*η*)	Small	-	Yes	With increasing *η*
Viscosity (*μ*)	Very Small	-	Yes	With decreasing *μ*
Friction Coefficient (*β*_SL_)	Very Small	-	Yes (only for high viscosities ≥ 5 mPa·s)	With decreasing *β*_SL_

## Data Availability

Data available on request.

## Supplementary Materials

The following supporting information can be downloaded at: https://www.mdpi.com/article/10.3390/ma16237284/s1, Section S1: effect of contact angle, EW number and viscosity on the droplet’s oscillation period during EW-induced detachment; Figure S1: droplet oscillation period under different EW numbers, contact angles, and viscosities; Section S2: parameters that affect the droplet’s ability to detach; Figure S2: number of pulses required for the detachment of a 5 μL droplet with a viscosity of 5 mPa·s, for different contact angles and EW numbers; Section S3: energy analysis; Figure S3: energy analysis of non-optimally synchronized EW; Figure S4: energy conversion at maximum elevation, for droplets of different volumes; Figure S5: energy conversion at maximum elevation, for droplets of different densities; Figure S6: energy conversion at maximum elevation, for droplets of different surface tensions; Video S1: detachment of a 5 μL water droplet (with *θ*_Y_ = 116°) using a single pulse (*η* = 0.51, *T*_p_ = *T*_s_ = 6.4 ms) (Figure 6); Video S2: detachment of a 5 μL water droplet (with *θ*_Y_ = 116°) using two pulses (*η* = 0.33, *T*_p_ = *T*_s_); Video S3: detachment of a 5 μL water droplet (with *θ*_Y_ = 116°) using three pulses (*η* = 0.25, *T*_p_ = *T*_s_); Video S4: detachment of a 5 μL water droplet (with *θ*_Y_ = 116°) using four pulses (*η* = 0.20, *T*_p_ = *T*_s_) (Figure 7).
