# Supplementary material for: Simulation of Electrowetting-Induced Droplet Detachment: A Study of Droplet Oscillations on Solid Surfaces
Source: Materials (Basel). 2023 Nov 23;16(23):7284. doi: 10.3390/ma16237284 (PMC10707388; doi:10.3390/ma16237284)
Supplement: Supplementary file 1 [file materials-16-07284-s001.zip › Supplementary_Material.pdf]

materials

Article

Simulation of Electrowetting – Induced Droplet Detachment: A Study of Droplet Oscillations on Solid Surfaces

Supplementary Material

Nicolas T. Theodorou [1], Alexandros G. Sourais [1] and Athanasios G. Papathanasiou [1,*]

[1] School of Chemical Engineering, National Technical University of Athens, 15780 Athens, Greece; nikolaostheodorou@mail.ntua.gr (N.T.T.); alexandrossourais@mail.ntua.gr (A.G.S.)
* Correspondence: pathan@chemeng.ntua.gr; Tel.: +30-210-772-3234

S1. Effect of Contact Angle, EW Number and Viscosity on the Droplet's Oscillation Period during EW – Induced Detachment

As mentioned in section 3.2.2 of the main article, the dependence of the droplet's oscillation period, prior to detachment, on its Young's contact angle (θ_Y), EW number (η) and viscosity (μ) was examined. The simulation's results are presented in Figure 9 of the main article. Additional results are displayed in Figure S1 below.

Wang et al. [1] have reported that the resonance frequency (ω) of a droplet scales proportionally with $[\gamma R_o^{-3}(6 - \eta)/(3\rho_w + 2\rho_a)]^{1/2}$, where R_0 is the droplet's equivalent radius, η is the EW number, ρ_w is the density of the droplet and ρ_a is the density of the ambient phase – Equation (33) in main article.

The simulation results, displayed in Figure 9 of the main article, are being compared to this theory in Figure S1 (a). The results have been fitted with Equation (S1):

$$T = C \cdot 2\pi \left[\gamma R_0^{-3} (6 - \eta) / (3\rho_w) \right]^{-1/2} \quad \text{(S1)}$$

where T is the droplet's oscillation period and C is a constant. This equation is derived from Equation (33) for an ambient phase of low density (e.g. air).

Both the simulation and the theoretical model [1] indicate that, for high EW numbers, the droplet's oscillation period increases with an increase of the EW number. However, for smaller EW numbers, simulations show that the oscillation period initially decreases with an increase of the EW number, forming a local minimum. This behavior is not predicted by the theoretical model. Nevertheless, the effect of the EW number on the droplet's oscillation period is small.

As results show in Figure S1, viscosity has a very small effect on the droplet's oscillation period. Vo et al., have analyzed the forces applied on the TPL of droplets under EW actuation, after splitting them in to two main categories: overdamped and underdamped [2]. Overdamped droplets approach their equilibrium contact radius monotonically, while underdamped droplets consistently overshoot it, while oscillating. In both regimes, the driving force that spreads the droplet is the electric force (F_{el}) at its TPL, which pulls the droplet horizontally. In the overdamped regime, the driving force is opposed by the friction between the droplet and the substrate (F_{ct}), which depends on their in-between friction coefficient and the droplet's viscosity and velocity. Therefore, the droplet's oscillation period is affected by the electric (F_{el}) and friction (F_{ct}) forces. In the underdamped regime, viscosity is negligible and the driving force is resisted by the droplet's inertia. Thus, the droplet's oscillation period depends on the electric force (F_{el}) and the droplet's inertia (ρV). In the simulations shown in Figure S1, there was no friction between the droplet and the substrate ($\beta_{SL} = 0$). Therefore, the droplet falls in the underdamped regime and the influence of viscosity on the droplet's oscillation period is almost negligible.

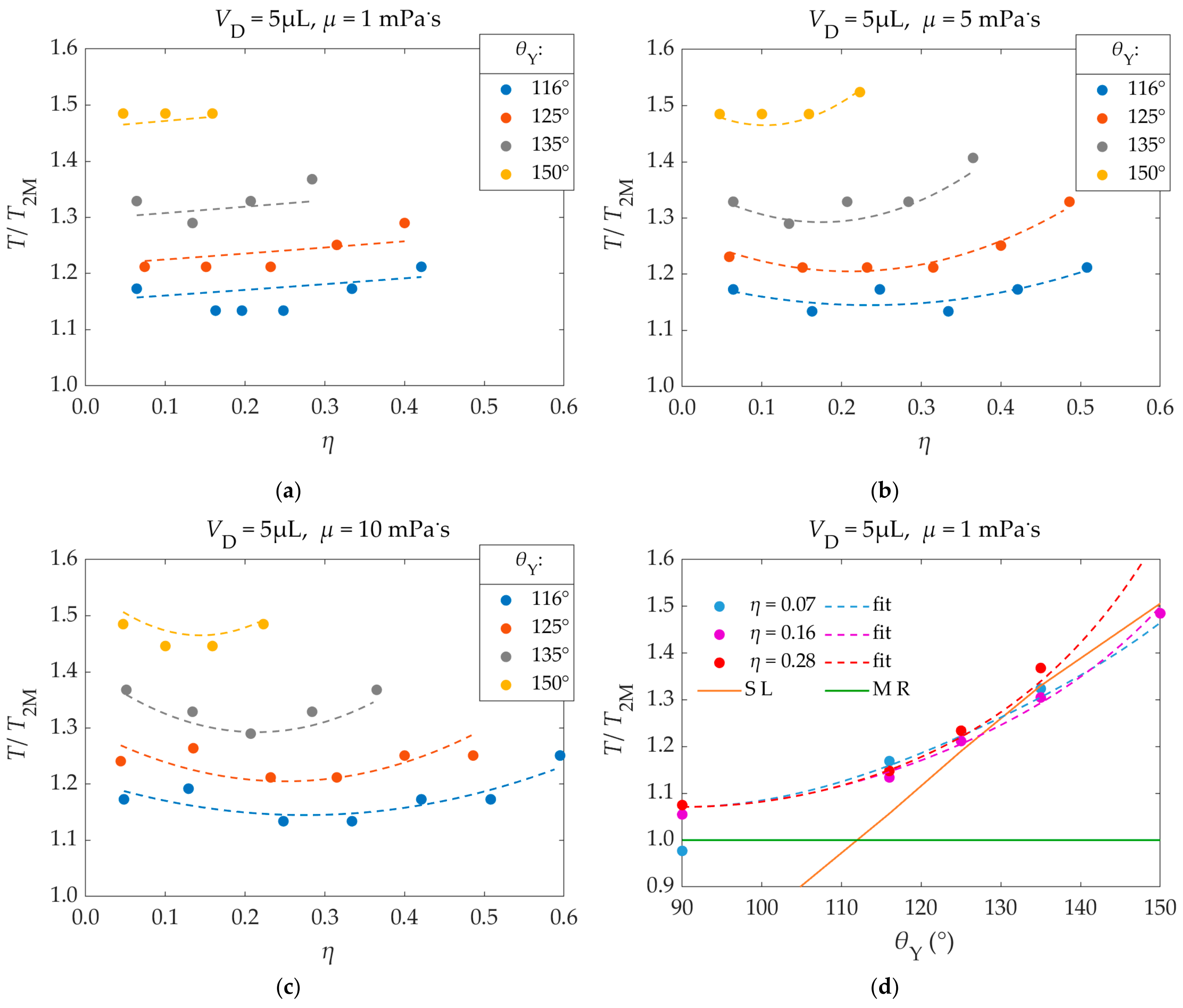

Figure S1. (**a**), (**b**), (**c**): Dimensionless oscillation period (T/T_{2M}) of a 5μL droplet's center of mass, as a function of the EW number (η) for different Young's Contact Angles (θ_Y) and a viscosity of (**a**) 1mPa·s, (**b**) 5mPa·s and (**c**) 10mPa·s. (**d**): Dimensionless oscillation period of the droplet as a function of Young's Contact Angle for different EW numbers and a viscosity of 1mPa·s. In all cases T_{2M} = 12.8 ms, ρ = 1000kg/m³, γ_{LA} = 72 mN/m and β_{SL} = 0. Points: Results, Dashed Lines: Fitting, Orange Line: Sakakeeny & Ling's model results (S L) [3], Green Line: Mirrored Rayleigh Droplet (M R). In case (**a**) the results have been fitted with the theoretical model [1] – Equation (S1). The fitting coefficients are C = 0.814, 0.859, 0.917, 1.032 for θ_Y = 116°, 125°, 135°, 150° respectively. In cases (**b**), (**c**), (**d**) the results have been fitted with Equation (32).

Hong et al. [4] have developed an equation that can predict the damped oscillation period of a droplet, under the influence of viscosity. Their model was validated with the results of droplet simulations with viscosities ranging from 1.0 up to 219.0 mPa·s. The damped oscillation period is given by the following equation:

$$\tau_d = \frac{1}{\sqrt{1-\zeta^2}}\tau_n \quad \text{, where}$$
$$\zeta = \left[\frac{(2n+1)}{n(n+2)}\mu + \frac{A_{nm}}{(n-1)(n+2)}\lambda\right]\sqrt{\frac{n(n-1)(n+2)}{\rho R_0 \gamma}} \tag{S2}$$

In this equation, τ_d is the oscillation period of the damped droplet, τ_n is the oscillation period of the non-damped droplet (without any friction or viscosity) and ζ is the damping factor that corresponds to the Ohnesorge (*Oh*) number, which represents the ratio of the viscous and frictional forces to the inertial and surface tension forces. The parameters on which ζ depends are the following: mode of oscillation (n), droplet viscosity (μ), parameter of the droplet's shape mode (A_{nn}), friction coefficient between the droplet and the substrate (λ), droplet density (ρ), droplet's equivalent radius (R_0), droplet's liquid – ambient phase surface tension coefficient (γ).

In the executed simulations shown in Figure S1, there was no friction between the droplet and the substrate (β_{SL} = 0). Therefore λ = 0. The ratios between the period of the damped and the non-damped droplet oscillation (τ_d/τ_n) that result from Equation (S2) are 1.00002, 1.00051 & 1.00205 for droplets of viscosities μ = 1, 5 & 10 mPa·s respectively (Parameters: n=2 (dominant mode), ρ = 1000 kg/m^3, V = 5μL, R_0 = 1.06 mm, γ = 72mN/m). Therefore, the oscillation periods of the high viscosity droplet (10mPa·s) are larger than those of the low viscosity droplet (1mPa·s) only by 0.2%, according to the model. Thus, the difference between them is negligible. This complies with the simulation results.

S2. Parameters that Affect the Droplet's Ability to Detach

As mentioned in section 3.3 of the main article, the number of voltage pulse applications (N) that are required for the detachment of a 5μL droplet was examined under different Young's contact angles (θ_Y), EW numbers (η) and viscosities (μ). The results concerning droplet viscosities of 1 mPa·s and 10 mPa·s were shown in Figure 10 of the main article. The results for a droplet with a viscosity of 5 mPa·s are shown in Figure S2. In this figure, the number of required pulses (N) for droplet detachment is displayed as a function of Young's contact angle (θ_Y) and the EW number (η). Pulses were synchronized with the droplet's spreading phase.

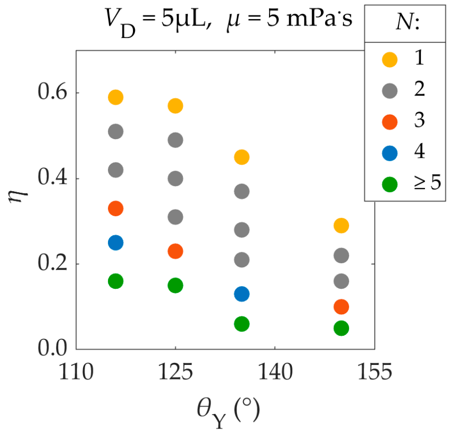

Figure S2. Number of voltage pulses (N) that are required for the detachment of a 5μL droplet, as a function of Young's contact angle (θ_Y) and the EW number (η), for a viscosity of μ = 5mPa·s. The density, surface tension and solid – liquid friction coefficient of the droplet are ρ = 1000kg/m^3, γ_{LA} = 72 mN/m and β_{SL} = 0 respectively.

S3. Energy Analysis

As mentioned in the main article, an energy analysis was made in order to better understand the phenomenon of electrowetting – induced detachment and the way in which energy is being transferred inside the droplet during the application of voltage.

S3.1. Energy Analysis of Optimally Synchronized EW – induced Droplet Detachment

The detachment of a 5μL water droplet from a substrate of $\theta_Y = 116°$, using two consecutive electric pulses of $\eta = 0.33$, synchronized with the droplet's spreading phase ("optimal synchronization"), was studied. The different energy forms that arise through the process are presented in Figure 12 (a) of the main article.

As shown in Figure 12 (a), the Accumulated Electric Work (W_{el}) provided to the droplet during the application of voltage, gets converted to Kinetic energy (K), Surface energy (E_S), Gravitational energy (U_G), as well as viscous dissipation (E_V), while the total energy of the system remains constant according to the energy balance – Equation (14).

Thanks to the synchronization between the electric pulse and the droplet's spreading phase, the electric power is mostly positive, as the electric force that spreads the droplet has the same direction with the velocity of the spreading droplet's TPL (they both point towards the outside of the droplet). This is depicted in Figure 12 (a), as an increase of the Accumulated Electric Work (W_{el}). However, due to small delays that are involved in the estimation of the TPL's velocity and the activation/deactivation of the simulated pulse, for a small amount of time, after the droplet has reached its maximum spreading radius, the electric power becomes negative. In these cases, the droplet starts to retract while voltage has not been removed yet. Hence, the electric force pulling the droplet and the droplet's TPL velocity point towards opposite directions. This is depicted in Figure 12 (a), as peaks in the curve of the Accumulated Electric Work (W_{el}), where the accumulated work starts to drop for a short period of time. When voltage is not applied the accumulated work remains constant, as no work is provided to the droplet. Therefore, the curve of the Accumulated Electric Work increases in the form of steps, where each step corresponds to an applied pulse. In the case of Figure 12 (a), two pulses were applied prior to droplet detachment, thus the curve of the Accumulated Electric Work rises in two steps.

Depending on the status of the droplet, the distribution of the droplet's energy into Kinetic, Gravitational and Surface Energy changes continuously over time. The droplet's Surface Energy reaches a maximum value when the droplet is fully spread on the substrate (thanks to the applied pulse). The surface energy is minimized when the shape of the droplet is similar to that of the initially resting droplet, since the equilibrium state of the droplet in the absence of voltage is that of minimum surface energy. This is mostly visible when the droplet reaches its initial radius, during its retraction, while its contact angle with the substrate is equal to the Young contact angle in the absence of voltage. When the droplet's contact radius becomes smaller than that of the resting stage and when the droplet detaches, its surface energy is larger than minimum, but not as large as that of the fully spread droplet prior to detachment.

The Gravitational Potential Energy of the droplet is proportional to the elevation of its center of mass. Therefore, it reaches a local maximum value when the droplet is fully retracted in each oscillation and a global maximum when the droplet's center of mass attains its maximum elevation after detachment. The droplet's gravitational energy is minimized at the points where the droplet is fully spread, due to pulse application.

The Kinetic Energy of the droplet is high when the liquid that constitutes the droplet is moving and zero when the droplet is resting. The droplet's kinetic energy reaches minimum values at the highest and lowest points of each oscillation, where the droplet becomes almost motionless. The kinetic energy gets larger as the droplet accelerates and reaches its maximum value when the contact radius of the droplet is close to its initial value, when the droplet was resting.

After the detachment of the droplet, a frequent exchange between its Kinetic Energy and its Surface Energy can be seen in Figure 12 (a), where peaks in the curve of the Kinetic Energy and wells of the same amplitude in the curve of Surface Energy, appear at the exact same times. This behavior is caused by the oscillations of the free airborne droplet that change the shape of its surface over time. By performing a FFT on the droplet's Surface Energy and Kinetic Energy as a function of time, it was found that their values oscillate with a dominant period of T_{1E} = 5.75ms and a secondary period of T_{2E} = 2.23ms. After multiplying these values by 2, since the Kinetic and Surface Energy oscillate two times within a single oscillation of the droplet, it can be seen that they are very close to the oscillation periods of the first two modes (n=2, n=3) of the free droplet, which are equal to T_2 = 9.05ms and T_3 = 4.67ms, according to Rayleigh & Lamb's equation – Equation (29).

Due to the viscous forces inside the droplet, the Electric Work that is given to the droplet does not get converted only to the useful forms of energy (Kinetic, Gravitational and Surface Energy), but also into viscous dissipation. As shown in Figure 12 (a), viscous dissipation only increases with time, since it is the accumulated work of frictional and viscous forces. Frictional forces mainly develop at the droplet's TPL. Viscous dissipation increases significantly, each time voltage is being removed, since the droplet's shape near the TPL changes rapidly, transitioning from the apparent contact angle (θ_a) (equal to 96° in this case – derived from Lippmann's Equation – Equation (8)) to the Young contact angle (θ_Y) in the absence of voltage (equal to 116° in this case). When the droplet is airborne, after its detachment, viscous dissipation is relatively low. At the moment where the droplet reattaches to the substrate, viscous dissipation increases significantly.

S3.2. Energy Analysis of Non-Optimally Synchronized EW

An energy analysis was performed in two scenarios where the applied pulse was not synchronized with the droplet's spreading phase. Both of these scenarios included the application of a double square pulse of η = 0.33 on a 5μL water droplet, as in S3.1. However, the pulse duration and the time interval between consecutive pulse applications was different (non – optimal). In the first scenario, pulse durations were shorter than optimal (T_P = 0.9T_s = 5.76 ms), while in the second one pulse durations were longer than optimal (T_P = 1.35T_s = 8.64 ms). In both scenarios the period between consecutive pulse applications was double the corresponding pulse duration T_t = 2T_P. The spreading time of a 5μL water droplet was previously found to be equal to T_s = 6.4 ms (section 3.1 – main article). The droplet did not detach from the substrate in any of these scenarios. The different forms of energy that appear during the process are presented in Figure S3. In both cases, less energy is given to the droplet compared to the "optimally synchronized" scenario of Section S3.1 (Figure 12 (a) – main article).

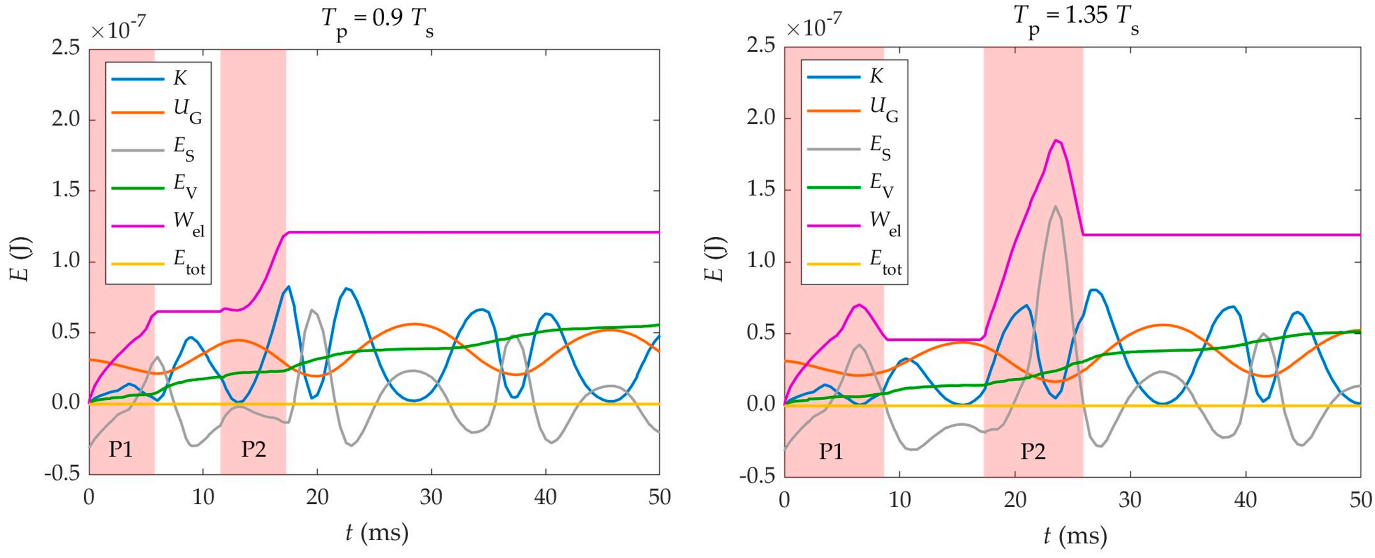

(**a**) (**b**)

Figure S3. Energies of a 5μL water droplet, during the process of non – optimally synchronized EW, as a function of time. Two consecutive square pulses of $\eta = 0.33$ were applied. The application times of the pulses (P1, P2) are marked as red patches in the background. Pulse widths: (**a**) $T_P = 0.9T_S$, (**b**) $T_P = 1.35T_S$. Period between consecutive pulse applications: $T_t = 2T_P$. Spreading time: $T_s = 6.4$ ms. The energies shown are Kinetic Energy (K), Gravitational Potential Energy (U_G), Surface Energy (E_S), Accumulated Viscous Dissipation (E_V), Accumulated Electric Work (W_{el}) and Total Energy (E_{tot}). There is no friction between the droplet and the substrate ($\beta_{SL} = 0$).

As shown in Figure S3 (a), when the applied pulse's duration and period are shorter than optimal, the Accumulated Electric Work given to the droplet is smaller. Although the EW number is the same (η=0.33) with the optimal case of Figure 12 (a), leading to the application of the same electric force, the force's application time is shorter. Thus, the droplet moves less during the application of the pulse, resulting in a smaller Electric Work. This is seen in Figure S3 (a), as smaller rises in the curve of the Accumulated Electric Work (W_{el}) during pulse application, when compared to the optimal scenario. Also, when the second pulse is applied to the droplet, the latter hasn't fully retracted. This leads to the Electric Work being negative for a very short period of time, as the electric force and the droplet's TPL velocity, point into opposite directions. This appears in Figure S3 (a) as a very small well in the curve of the Accumulated Electric Work (W_{el}), at the beginning of the second pulse's application.

In the case where the pulse duration is longer than optimal, the Accumulated Electric Work at the end of the EW process is again smaller when compared to the optimal case. Similarly, to the optimal case, pulses are being applied during the spreading phase of the droplet, initially giving the same amount of work. However, unlike the optimal scenario, pulses are still being applied at the beginning of the droplet's retraction. This leads to the Electric Work being negative for short periods of time, after the droplet has reached its maximum spreading radius in each oscillation. The negative Electric Work is a result of the spreading electric force and the velocity of the droplet's TPL pointing in different directions during the droplet's retraction. This phenomenon is depicted in Figure S3 (b) as peaks of the Accumulated Electric Work (W_{el}), where the latter starts to get smaller when the droplet retracts, while pulses are still applied. Thus, the Accumulated Electric Work at the end of each pulse application is smaller than that of the optimal case.

In both scenarios, the smaller amount of given work, leads to the droplet being unable to detach, unlike the case were the pulses were synchronized with the droplet's spreading phase (Section S3.1, Figure 12 (a) – main article).

S3.3. Energy Analysis on Water Droplets of Different Volumes

An energy analysis was made on water droplets of different volumes (V = 1, 3, 5 & 10 μL), that detached after the application of double square pulses of η = 0.33. Pulses were synchronized with the droplets' spreading phase. These scenarios were previously presented in section 3.2.1 of the main article.

In each scenario, the point of maximum droplet elevation was determined, by locating the timeframe where the droplet's gravitational potential energy reached its maximum value. At this point the values of each different energy form and their increase compared to the beginning of the simulation were recorded for each droplet volume. The results are presented in Figure S4 (a) in a log – log diagram. The increase of each energy is also plotted as a percentage of the provided electric work in Figure S4 (b).

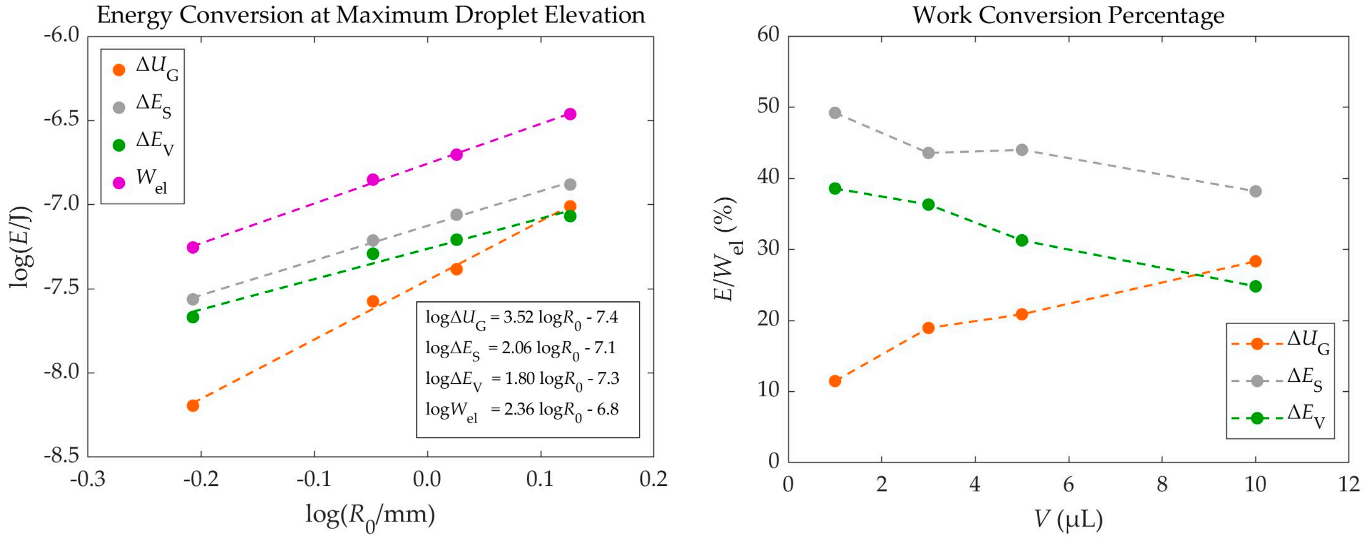

(**a**) (**b**)

Figure S4. (**a**) Increase of different energies at the point of maximum droplet elevation as a function of the droplet's equivalent radius ($R_0 = (3/(4\pi)V)^{1/3}$). The energies shown are Gravitational Potential Energy (ΔU_G), Surface Energy (ΔE_S), Accumulated Viscous Dissipation (ΔE_V) and Accumulated Electric Work (W_{el}). (**b**) Percentages of the different energy forms that the electric work, given to the droplet during the EW process, is being converted to, as a function of droplet volume. The sum of percentages is less than 100%, as a small percentage of converted energy remains as kinetic energy (not shown). All droplets were made of water (μ = 1mPa·s, ρ = 1000kg/m^3, γ = 72 mN/m). Every droplet detached after the application of a double pulse of η = 0.33. In all cases θ_Y=116°, β_{SL} = 0.

As shown in Figure S4 (a), it can be seen that the Gravitational Potential Energy of the Droplets is proportional to $R_0^{3.5}$. This value is close to 3 and makes sense, since if all droplets reached the same elevation after detachment, their gravitational energy would be proportional to their mass, which is proportional to their volume that is proportional to R_0^3. The deviation from the number of 3 could be a result of dispersion of the simulation's results. The fact that the gravitational energy scales almost proportionally with R_0^3, indicates that the Volume of the droplet does not affect significantly its ability to detach.

The Surface Energy and the Accumulated Electric Work given to the droplet at the peak of each trajectory scale linearly with R_0^2. This is the result of the free droplet's surface energy being proportional to its surface and the electric work being proportional to the increase of the contact area between droplet and the substrate. So, although more energy is required for the detachment of larger droplets (gravitational, surface, viscous dissipation), it is provided, thanks to their larger surface.

The Accumulated Viscous Dissipation scales linearly with $R_0^{1.80}$. The exponent 1.80 is relatively close to 1. This makes sense, since the viscous dissipation during EW actuation is mostly developed at the droplet's TPL, making it proportional to $2\pi R_0$. It is greater than 1, as viscous dissipation is also developed on the inside volume of the droplet.

Figure S4 (b) shows that the percentage of the Electric Work given to the droplet that gets converted into Viscous Dissipation, gets lower with an increase of the droplet's volume, making the detachment process more efficient, from an energy perspective.

S3.4. Energy Analysis on 5μL Droplets of Different Densities

An energy analysis was also made on 5μL droplets of different densities (500, 1000 & 2000 kg/m³), that detached after the application of a double pulse of η = 0.33. Pulses were synchronized with the droplets' spreading phase. These scenarios were also presented in section 3.2.1 of the main article.

The amounts of each energy at the droplet's maximum elevation, that the electric work provided to the droplet gets converted to, are plotted for each droplet in Figure S5 (a). The energies are also plotted as a percentage of the electric work in Figure S5 (b).

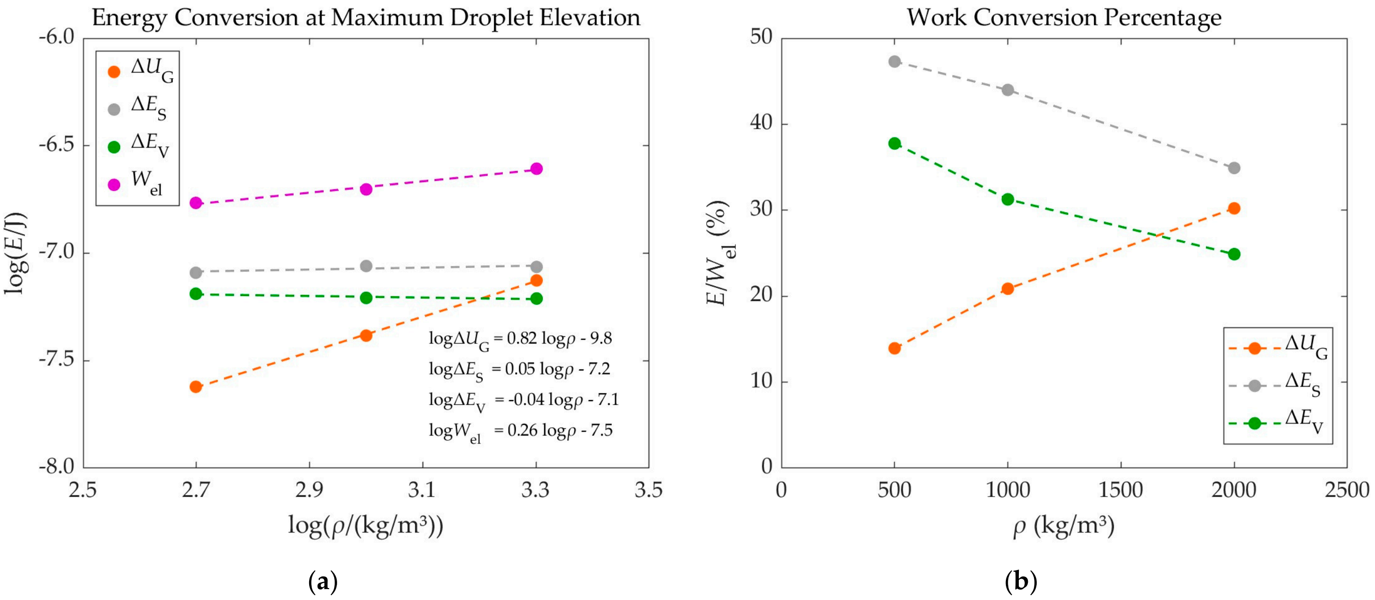

Figure S5. (**a**) Increase of different energies at the point of maximum droplet elevation as a function of the droplet's density (ρ). The energies shown are Gravitational Potential Energy (ΔU_G), Surface Energy (ΔE_S), Accumulated Viscous Dissipation (ΔE_V) and Accumulated Electric Work (W_{el}). (**b**) Percentages of the different energies that the provided electric work is being converted to, at maximum droplet elevation, as a function examined droplet's density. The sum of percentages is less than 100%, as a small percentage of converted energy remains as kinetic energy (not shown). The droplets' viscosity and surface tension were μ = 1mPa·s and γ = 72 mN/m. Every droplet detached after the application of two consecutive pulses of η = 0.33. In all cases θ_Y=116°, β_{SL} = 0.

In Figure S5 (a), it can be seen that the droplet's Surface energy and Viscous Dissipation are almost independent of the droplet's density. This makes sense, since all droplets have the same volume (V=5μL) and therefore similar surface and radius.

The Gravitational Energy scales linearly with $\rho^{0.82}$. This is close to being proportional with ρ, which is the case when all droplets reach the same elevation after detachment, since their gravitational energy is proportional to their mass, hence their density (V=5μL for all droplets). This indicates that the droplet's ability to detach is practically independent of its density.

The Accumulated Electric Work, scales with the droplet's density as $\rho_0^{0.26}$. All the droplets have the same volume (V=5μL) and therefore it would be expected that their contact surface with the substrate would be the same, leading to the same electric work. However, larger droplet densities lead to larger droplet mass and inertia. As a result, dense droplets tend to overshoot their equilibrium contact radius more, while oscillating on the substrate, due to their larger inertia. This leads to a slightly larger variation of the surface between the droplet and the substrate and causes the Electric Work that is given to the denser droplets to be larger. So, although, more gravitational energy is required for the detachment of denser droplets, it is given to them, thanks to their slightly larger solid – liquid interface.

Similarly, to the case of different tested volumes, in Figure S5 (b) it can be seen that, as the density of the droplet gets larger, the percentage of the given electric work that is converted to viscous dissipation is smaller.

S3.5. Energy Analysis on 5μL Droplets with Different Surface Tension Coefficient

An energy analysis was made as well on 5μL droplets, with different liquid – ambient phase surface tension coefficients (γ = 36, 72, 108, 144 & 180 mN/m), that detached after the application of two consecutive pulses of η = 0.33. The pulses were synchronized with each droplet's spreading phase. These scenarios are also presented in section 3.2.2 of the main article.

The amounts of each energy at the droplet's maximum elevation, that the electric work gets converted to, are plotted for each droplet in Figure S6 (a). These energies are also plotted as a percentage of the provided electric work in Figure S6 (b). The Kinetic Energy is also plotted in this figure, as its presence is significant in some cases.

The larger the surface tension, the higher was the jump of the droplet. Another scenario where γ = 18 mN/m was tested and the droplet did not manage to detach under the same EW number. This indicates that the surface tension coefficient affects the droplet's ability to detach.

The Gravitational Energy of each droplet at maximum elevation scales linearly with $\gamma^{0.75}$. Since all of the examined droplets had the same mass, the fact that the gravitational energy increases with an increase of the surface tension coefficient, shows that the droplets perform a higher jump at higher surface tensions and detach more easily.

The Electric Work and the Surface Energy of the droplets scale linearly with γ. This makes sense, as both of these energies are proportional to the surface tension coefficient of the droplet. All droplets have the same volume and the same surface.

Viscous dissipation scales linearly with γ. Since the viscous dissipation is mainly generated on the droplet's TPL, it is significantly affected by the droplet's surface tension coefficient.

As shown in Figure S6 (b), the percentage of the electric work that is converted to viscous dissipation increases slightly with an increase of the surface tension coefficient, although the droplet detaches more easily and jumps higher. This is a result of the excess energy given to the droplet in case of the higher surface tensions, that leads to a faster movement of the droplet's TPL and higher viscous forces due to higher velocity gradients.

The Kinetic and Surface Energy of the droplet present an oscillatory behavior for different surface tensions. This is a result of the droplet being at a different phase of its free oscillations, when it reaches its maximum elevation after detachment, in each scenario.

It can be concluded that a change in the surface tension coefficient (γ) of a droplet, while all other parameters are maintained (θ_Y, η, V, ρ, μ), affects its ability to detach. Droplets detach more easily for higher surface tension coefficients, although the difference is relatively small.

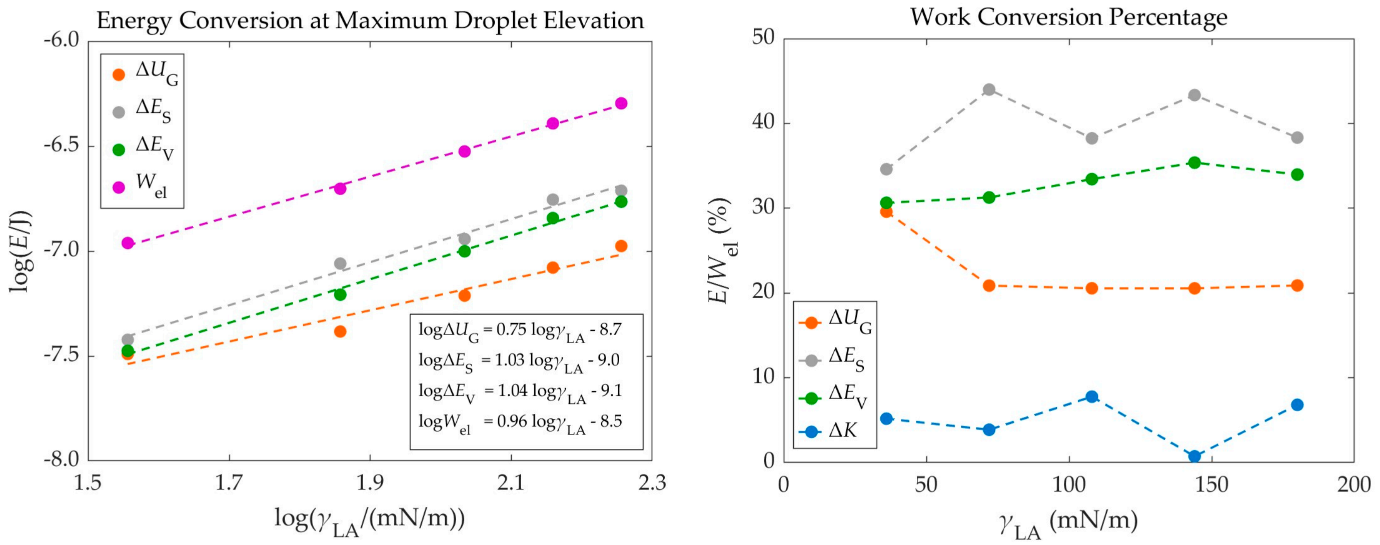

(**a**) (**b**)

Figure S6. (**a**) Increase of different energies at the point of maximum droplet elevation, compared to the beginning of the simulation, as a function of the droplet's liquid – ambient phase surface tension coefficient (γ_{LA}). (**b**) Energies as percentages of the provided electric work. The energies shown are Gravitational Potential Energy (ΔU_G), Surface Energy (ΔE_S), Accumulated Viscous Dissipation (ΔE_V), Accumulated Electric Work (W_{el}) and Kinetic Energy (ΔK). The droplets' viscosity and density were μ = 1mPa·s & ρ = 1000kg/m^3. Every droplet detached after the application of a double pulse of η = 0.33. In all cases θ_Y=116°, β_{SL} = 0.

S3.6. Energy Analysis on Droplet Detachment Using a Different Number of Pulses

As mentioned in section 3.4 of the main article, an energy analysis was made on four different scenarios, where a 5μL water droplet detached from a solid surface after the application of N = 1, 2, 3 & 4 pulses that corresponded to EW numbers equal to η = 0.51, 0.33, 0.25 & 0.20 respectively. In each scenario, all pulses were of the same EW number. In all Young's contact angle was equal to 116°. This analysis was made in order to determine the most efficient method for detaching the droplet, from an Energy perspective, among the use of a single pulse or multiple pulses.

The Electric Work given to the droplet in each scenario, as well as the different Energy forms that it gets converted to at the droplet's maximum elevation, are shown in Figure 12 (b) in the main article.

The energy analysis of Figure 12 (b) shows that, when the 5μL droplet gets barely detached from the substrate (using an EW number close to threshold), its Kinetic, Gravitational and Surface Energy (useful forms of energy) at the maximum elevation reached, are independent of the number of pulses (N) that are used in the detachment process. However, the Electric Work and the Viscous Dissipation inside the droplet become larger, as the number of pulses (N) increases.

Therefore, although using more pulses reduces the threshold EW number and the required voltage for detachment, it increases the amount of required energy. This means that the more pulses are used for detachment, the less efficient the process is from an energy perspective, since more electric energy is spent, while the droplet detaches and reaches the same height.

References

1. Wang, Z.; Ende, D. van den; Pit, A.; Lagraauw, R.; Wijnperlé, D.; Mugele, F. Jumping Drops on Hydrophobic Surfaces, Controlling Energy Transfer by Timed Electric Actuation. *Soft Matter* **2017**, *13*, 4856–4863, doi:10.1039/C7SM00928C.
2. Vo, Q.; Su, H.; Tran, T. Universal Transient Dynamics of Electrowetting Droplets. *Sci Rep* **2018**, *8*, 836, doi:10.1038/s41598-018-19167-7.
3. Sakakeeny, J.; Ling, Y. Natural Oscillations of a Sessile Drop on Flat Surfaces with Mobile Contact Lines. *Phys. Rev. Fluids* **2020**, *5*, 123604. https://doi.org/10.1103/PhysRevFluids.5.123604.
4. Hong, J.; Kim, Y.K.; Kang, K.H.; Oh, J.M.; Kang, I.S. Effects of Drop Size and Viscosity on Spreading Dynamics in DC Electrowetting. *Langmuir* **2013**, *29*, 9118–9125, doi:10.1021/la401801u.
